# Metatranscriptomic Analysis of Tick Virome Diversity in Hebei Province, China

**DOI:** 10.3390/v18040443

**Published:** 2026-04-07

**Authors:** Minghao Geng, Xueqi Wang, Xiaoxia Huang, Yan Li, Yamei Wei, Yanan Cai, Jiandong Li, Caixiao Jiang, Wei Wu, Shiyou Liu, Nana Guo, Xinyang Zhang, Wentao Wu, Guangyue Han, Xu Han, Tiezhu Liu, Qi Li, Shiwen Wang

**Affiliations:** 1 National Key Laboratory of Intelligent Tracking and Forecasting for Infectious Diseases, National Institute for Viral Disease Control and Prevention, Chinese Center for Disease Control and Prevention, Beijing 102206, China; gmhcdc@126.com (M.G.); huangxx@ivdc.chinacdc.cn (X.H.); ldong121@126.com (J.L.); wuwei@ivdc.chinacdc.cn (W.W.); 2 Institute for Viral Disease Prevention and Control, Hebei Provincial Center for Disease Prevention and Control, Shijiazhuang 050000, China; lian.2002@163.com (Y.L.); weiyamei2013@163.com (Y.W.); yanan589@163.com (Y.C.); jiangcaixiao@163.com (C.J.); lsy7@outlook.com (S.L.); yufeiwet@163.com (N.G.); zxhuade@126.com (X.Z.); wuwentao@zju.edu.cn (W.W.); hanguangyue2013@163.com (G.H.); hbcdchanxu@163.com (X.H.); 3 Capital Center for Children’s Health, Capital Medical University, Capital Institute of Pediatrics, Beijing 102206, China; iwangxueqi@gmail.com; 4 Hebei Key Laboratory of Pathogens and Epidemiology of Infectious Diseases, Hebei Provincial Center for Disease Control and Prevention, Shijiazhuang 050000, China

**Keywords:** virome, SFTSV, tick-borne disease, metatranscriptomic

## Abstract

Ticks serve as primary vectors for a wide array of RNA viruses, yet the diversity and distribution of tick-associated RNA viruses remain incompletely characterized in Hebei province. To address this gap, we conducted a systematic metatranscriptomic investigation of 986 ticks representing six species, collected from the diverse ecological landscapes of Hebei Province in northern China. Our analysis recovered 25 complete or near-complete viral genomes spanning 12 families, including *Phenuiviridae*, *Flaviviridae*, and *Nairoviridae*. Of critical public health significance, we identified Severe Fever with Thrombocytopenia Syndrome Virus (SFTSV) in both *Haemaphysalis longicornis* and *Dermacentor nuttalli*. Phylogenetic reconstruction revealed marked geographic stratification where strains from the coastal plains clustered with the dominant Genotype F, while those from the mountainous north formed a characteristic and divergent lineage phylogenetically linked to isolates from Inner Mongolia. Furthermore, a novel viral agent provisionally named Zhangjiakou Hepacivirus was discovered in *Haemaphysalis japonica*. This virus shared less than 80% nucleotide identity with the rodent-associated Hepacivirus P, consistent with a rodent origin and possible cross-species transmission. Collectively, these findings reveal descriptive variation associated with vector identity, physiological status, and ecological context in shaping viral evolution and underscore the need for continuous metagenomic surveillance to mitigate emerging tick-borne disease risks within a One Health framework.

## 1. Introduction

Ticks (Ixodoidea) [1], as obligate hematophagous ectoparasites, are significant vectors of pathogens worldwide, second only to mosquitoes in public health impact [2]. To date, over 100 tick-borne viruses (TBVs) have been identified globally, with nearly 60 known to be pathogenic to humans [3], including clinically important pathogens such as Crimean-Congo hemorrhagic fever virus (CCHFV) [4], tick-borne encephalitis virus (TBEV) [5] and severe fever with thrombocytopenia syndrome virus (SFTSV) [6]. Furthermore, the convergence of climate change, habitat fragmentation, and anthropogenic expansion has intensified human–tick interfaces, exacerbating the epidemic risk of these historically neglected natural focal diseases [7].

In recent years, surveillance efforts in China have led to the discovery of several novel tick-borne pathogens, including Alongshan virus [8], Orthonairovirus songlingense [9], Xuecheng orthonairovirus [10], and Wetland orthonairovirus [11]. These findings, often linked to unexplained febrile illness, suggest that the true diversity of the tick virome and its potential threat to public health remain largely underexplored [12]. Consequently, continuous and systematic surveillance of tick viromes is imperative for early warning and mitigation of emerging infectious diseases [13].

For the present investigation, we conducted tick sampling across three administrative regions of Hebei Province that span a range of landscapes: Zhangjiakou (ZJK), located on the Bashang Plateau in the northwest (elevation ~1200 m); Cangzhou (CZ), a low-lying coastal region adjacent to the Bohai Sea (elevation <20 m); and Shijiazhuang (SJZ), situated in the piedmont zone east of the Taihang Mountains (elevation ~200–300 m). These sites were selected to sample ticks across the province’s geographic breadth. Utilizing unbiased metatranscriptomic sequencing, we conducted a survey of tick-associated RNA viruses to catalog the diversity of known and novel viral agents and to phylogenetically characterize viruses of public health significance.

## 2. Materials and Methods

### 2.1. Sample Collection

**Figure 1:**
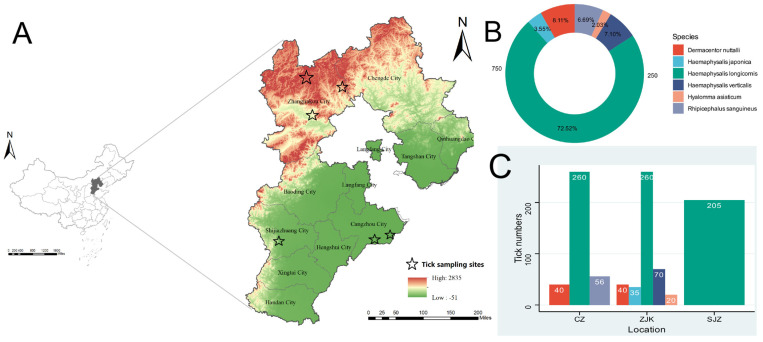
Sampling framework and tick species composition in Hebei Province, China. (**A**) Map showing the three regional groups sampled in this study: Zhangjiakou (ZJK), Cangzhou (CZ), and Shijiazhuang (SJZ). Stars indicate individual collection sites within each regional group. (**B**) Proportional composition of tick species among all collected specimens. (**C**) Numbers of each tick species collected in the three regional groups.

Ticks were collected in three regional groups in Hebei Province, China, namely Zhangjiakou (ZJK), Cangzhou (CZ), and Shijiazhuang (SJZ), from May to July 2024 (Figure 1). Within each regional group, one or more individual collection sites were sampled, as indicated in Figure 1. Sampling included forests and grasslands for free-living ticks, as well as domestic animals and small mammals for feeding ticks. Free-living ticks were collected by flagging vegetation with a 1 m^2^ flannel cloth, whereas feeding ticks were removed from hosts with sterile forceps. Ticks were collected as individual specimens in the field and were immediately transported under cold-chain conditions, then stored at −80 °C. In the laboratory, adult ticks were morphologically identified to species level by experienced entomologists and subsequently grouped into pools according to tick species, sampling region, and feeding status. Pool sizes ranged from 20 to 65 ticks per library. Each pool was processed as a single homogenized sample for nucleic acid extraction and library preparation. Because the primary objective of this study was exploratory virus discovery across multiple tick groups, pooled analysis was used to increase screening breadth.

### 2.2. RNA Library Construction and Sequencing

Collected ticks were rinsed three times with sterile phosphate-buffered saline (PBS) to remove surface contaminants and then homogenized at a low temperature. Homogenization was performed using a TissueLyser II (QIAGEN, Hilden, Germany) with 5-mm and 2-mm stainless-steel beads at 25 Hz for 10 min. The homogenates were centrifuged at 2000× *g* for 30 s, and the clarified supernatants were transferred to sterile microcentrifuge tubes. Total nucleic acids were extracted from the supernatants with a Viral Nucleic Acid Isolation Kit (Magnetic Beads; Jiangsu Shoushi Technologies Co., Ltd., Taizhou, China) according to the manufacturer’s instructions, and their concentrations were measured using a Qubit 4.0 fluorometer (Thermo Fisher Scientific, Eugene, OR, USA). Real-time RT-PCR assays for SFTSV RNA detection were performed using a commercial diagnostic kit (Guangzhou Daan Gene Technology Co., Ltd., Guangzhou, China) following the manufacturer’s protocol. RNA libraries were prepared with the VAHTS Universal V8 RNA-seq Library Prep Kit for Illumina (Vazyme, Nanjing, China) following the manufacturer’s protocol. The libraries were then sequenced on an Illumina NovaSeq 6000 platform (Illumina, San Diego, CA, USA) using paired-end reads (e.g., 2 × 150 bp) in accordance with the standard Illumina procedures.

### 2.3. Viral Contig Assembly and Annotation

The quality of raw sequencing data from each library was first assessed using FastQC v0.11.9 [14]. Adapter sequences and low-quality bases were then removed with Trimmomatic v0.39 [15], and the resulting high-quality reads were de novo assembled into contigs using MEGAHIT v1.2.9 [16] with default parameters. Assembly statistics were evaluated with QUAST v5.2.0 [17]. Assembled contigs were compared against the NCBI non-redundant protein (nr) database using DIAMOND v2.1 [18] BLASTx (e-value ≤ 1 × 10^−5^), and taxonomic assignments were inferred with MEGAN v6.24.9 [19]. Putative viral contigs were further examined by BLASTn [20] searches against the NCBI nucleotide (nt) database to confirm their viral origin. Clean reads were mapped back to viral contigs with Bowtie2 v2.3.5.1 [21], and viral abundance in each library was calculated as fragments per kilobase of contig per million mapped reads (FPKM) as well as raw read counts [22].

Because the extraction workflow recovered total nucleic acids and no DNase treatment was applied prior to library construction, residual DNA could not be completely excluded at the wet-lab stage. Therefore, in downstream analyses, we restricted interpretation to viral contigs assigned to established strains. For candidate novel viral sequences, open reading frames (ORFs) were predicted using NCBI ORFfinder, and conserved RNA-dependent RNA polymerase (RdRp) domains were identified by homology searches against the Conserved Domain Database (CDD) using NCBI’s CD-Search tool. Read-mapping profiles generated by Bowtie2 v2.3.5.1 [21] were used to examine coverage continuity and assess assembly integrity across each putative viral genome, ensuring robust identification of near-complete or complete genomes. Following commonly used metagenomic heuristics and published demarcation references, a virus was provisionally regarded as a candidate novel lineage if it shared <80% nucleotide identity across the available genome region or <90% amino acid identity within the RdRp region with its closest recognized relative, while acknowledging that formal species assignment is genus-dependent [23,24].

### 2.4. Phylogenetic and Evolutionary Analysis

Amino acid sequences of the viral RdRp domains were aligned with representative reference sequences from GenBank using MAFFT v7.490 [25]. Poorly aligned regions were trimmed using TrimAl v1.4 [26]. Maximum Likelihood (ML) phylogenetic trees were reconstructed using IQ-TREE v2.2.2.3 [27], employing the best-fit substitution model determined by ModelFinder [28]. Nodal support values were evaluated through 1000 ultrafast bootstrap replicates. To further assess sequence variation among pooled viral assemblies, nucleotide diversity was additionally evaluated for selected high-priority viruses using pairwise genetic distance analyses in MEGA11 [29]. Tree visualization and annotation were conducted using Chiplot (https://www.chiplot.online).

### 2.5. Statistical Analysis and Visualization

The Kruskal–Wallis test was conducted via the statannotations library within the Python 3.7 environment. Alpha diversity metrics, encompassing observed species richness and the Shannon diversity index, were computed using the vegan package in R 4.3.1 [30]. Alpha diversity was assessed using the Shannon diversity index (H′ = −Σp_i_ ln p_i_), which accounts for both species richness and evenness within each tick pool. For beta diversity analysis, pairwise Bray–Curtis dissimilarity matrices were calculated based on FPKM-normalized viral abundance profiles across all pools. Bray–Curtis dissimilarity ranges from 0 to 1 and was selected because it incorporates both compositional and abundance information. Ordination of the dissimilarity matrix was performed using principal coordinates analysis (PCoA) as implemented in the vegan package (v2.6) in R 4.3.1 [31]. To formally test whether tick species identity or sampling site significantly explained variation in virome composition, permutational multivariate analysis of variance (PERMANOVA) was performed using the adonis2 function in the vegan package (v2.6.4) in R 4.3.1, with 999 permutations on Bray–Curtis dissimilarity matrices. Heatmap visualization was accomplished utilizing the PyComplexHeatmap library [32]. Because pools differed in tick number and species composition, all diversity and abundance analyses should be interpreted as exploratory comparisons at the pooled-library level rather than estimates of individual-level infection burden.

## 3. Results

### 3.1. Sample Collection Results

A total of 986 ticks were collected from three regions of Hebei Province, China (Figure 1), between May and July 2024: Shijiazhuang (*n* = 205), Cangzhou (*n* = 356), and Zhangjiakou (*n* = 425) (Supplementary Table S1). The specimens comprised six species: *Haemaphysalis longicornis* (715 ticks, 73.0%), *Dermacentor nuttalli* (80 ticks, 8.1%), *Haemaphysalis verticalis* (70 ticks, 7.1%), *Rhipicephalus sanguineus* (66 ticks, 6.7%), *Haemaphysalis japonica* (35 ticks, 3.6%), and *Hyalomma asiaticum* (20 ticks, 2.0%). Species diversity varied among regions, where five species were identified in Zhangjiakou, three in Cangzhou, and only one in Shijiazhuang. *H. longicornis*, the dominant tick species in China, was ubiquitous across all three regions. Notably, *H. asiaticum* was detected in our survey of Hebei Province, warranting attention because of its epidemiological relevance as a recognized vector of CCHFV elsewhere. Tick species composition differed among the three regional groups. However, because environmental variables were not directly measured in this study, the drivers of these distributional patterns cannot be determined from the present dataset. The marked differences in altitude and precipitation among the three regions may be associated with the observed patterns of tick distribution. Ticks were further grouped into 23 pools according to species, sampling site, and blood-feeding status (Table S1).

### 3.2. Metatranscriptomic Sequencing and Viral Identification

**Figure 2:**
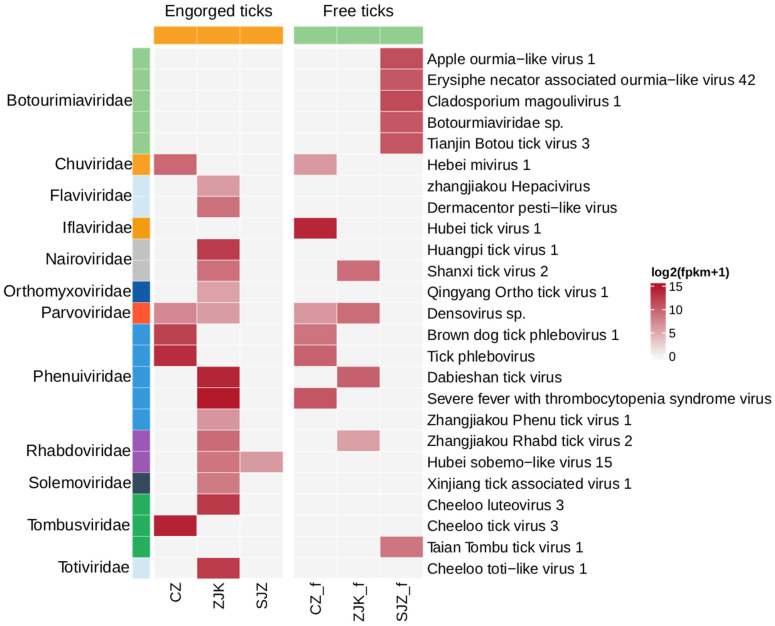
A heatmap of the abundances of viruses identified in the pools of free and engorged ticks collected from different locations. FPKM values were calculated by merging tick pools from the same location. Abbreviations: CZ, Cangzhou; ZJK, Zhangjiakou; SJZ, Shijiazhuang; suffix “_f” indicates free-living tick pools.

To characterize the tick virome, the 986 collected ticks were organized into 23 pools based on species, feeding status, and collection site (Cangzhou: 9; Zhangjiakou: 9; Shijiazhuang: 5), generating a total of 308.31 Gb of clean data. Each library yielded between 7.5 × 10^7^ and 1.2 × 10^8^ clean reads, containing 5.1 × 10^4^ to 6.6 × 10^5^ viral reads (Table S4). Based on ICTV criteria and a <90% RdRp amino acid identity threshold for novel species, we assembled complete or near complete-genomes for 25 viruses (Table S2) spanning 12 families, including *Botourimiaviridae*, *Chuviridae*, *Flaviviridae*, *Iflaviridae*, *Nairoviridae*, *Orthomyxoviridae*, *Parvoviridae*, *Phenuiviridae*, *Rhabdoviridae*, *Solemoviridae*, *Tombusviridae*, and *Totiviridae* (Figure 2). With *Phenuiviridae* and *Botourmiaviridae* exhibiting the highest abundance, relevant virus genome sequences have been deposited in the GenBase databases (Table S3). Notably, we identified a novel hepacivirus in engorged ticks from Zhangjiakou, tentatively named Zhangjiakou Hepacivirus, which shares close phylogenetic affinity with Hepacivirus P. Furthermore, to our knowledge, four strains of the virulent pathogen SFTSV were detected, representing the first identification of SFTSV in ticks from Hebei Province, China, and highlighting a potential public health risk. Due to its role as a primary vector for CCHFV, all *H. asiaticum* specimens were further screened for CCHFV using Real-time RT-PCR, which yielded negative results. To improve transparency of library-level viral composition, we summarized the mapped read counts for each detected viral taxon in each pooled library (Table S5). We summarized sequencing composition and alpha-diversity metrics for each pooled library in Supplementary Figure S1. This figure shows viral read counts, the proportion of viral reads among total clean reads, and the Shannon diversity and richness of detected viral taxa at the family and species levels. Although these metrics varied among pooled libraries, pairwise comparisons among the three regions did not show statistically significant differences for the displayed measures (*p* > 0.05). A total of 18 viral species were identified in tick pools from Zhangjiakou, compared with 7 from Cangzhou and 7 from Shijiazhuang.

### 3.3. Comparison of Detected Viral RNA Profiles Between Free and Engorged Tick Pools

To explore whether feeding status was associated with differences in detected viral RNA profiles, we compared pooled libraries from free and engorged ticks across the sampled regional groups. Heatmap visualization suggested different viral composition patterns between the two groups (Figure 2). In general, engorged tick pools showed higher total viral RNA abundance at the family level than free tick pools. Among the 25 identified viruses, eight were detected in both free and engorged tick pools. Several of these shared viruses, including Hebei mivirus 1, Shanxi tick virus 2, *Densovirus* sp., Brown dog tick phlebovirus 1, Tick phlebovirus, Dabieshan tick virus, SFTSV, and Zhangjiakou rhabdo tick virus 2, showed higher relative RNA abundance in engorged pools. However, these observations do not allow inference regarding within-tick replication, transmission potential, or vector competence.

**Figure 3:**
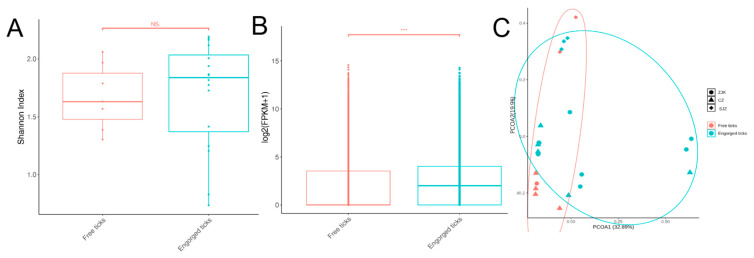
Comparison of viral diversity and abundance between free and engorged ticks. (**A**) Alpha diversity measured by the Shannon index for free ticks and engorged ticks. (**B**) Comparison of viral abundance expressed as log2(FPKM + 1) between free and engorged ticks. (**C**) Principal Coordinates Analysis (PCoA) based on Bray–Curtis dissimilarities of viral profiles. Each point represents a tick pool, with colors indicating feeding status (red: free ticks; teal: engorged ticks) and shapes indicating sampling regions (circles: Zhangjiakou, ZJK; triangles: Cangzhou, CZ; diamonds: Shijiazhuang, SJZ). Ellipses represent the 95% confidence intervals for each group. (***) indicate statistically significant differences (*p* < 0.05), (NS) indicates no statistically significant difference.

To characterize the impact of physiological status on the tick virome, we compared the viral profiles of free and engorged ticks. Alpha diversity analysis, measured by the Shannon index, showed no statistically significant difference between the two groups (Kruskal–Wallis, *p* > 0.05; Figure 3A), indicating that Shannon diversity did not differ significantly between pooled free and engorged tick libraries. In contrast, viral abundance exhibited a dramatic disparity. Analysis of viral transcript levels [log2(FPKM + 1)] revealed that engorged ticks possessed significantly higher viral abundance compared to free ticks (Kruskal–Wallis, *p* < 0.05; Figure 3B). This indicates that engorged tick pools contained higher relative viral RNA abundance than free tick pools. In the PCoA ordination, free tick pools showed relatively less dispersion compared to engorged tick pools (Figure 3C). However, PERMANOVA analysis did not detect a statistically significant difference in overall virome composition between the two groups (R^2^ = 0.06, *p* = 0.14). The wider dispersion of engorged tick pools in ordination space is consistent with the stochastic acquisition of diverse vertebrate host-derived viruses during feeding on different individual hosts, rather than a systematic shift in the tick-intrinsic virome. Metatranscriptomics of engorged ticks captures a composite signal of both tick-associated and host-derived viral RNA, as these ticks may acquire a variety of viral sequences from vertebrate blood meals in addition to harboring their intrinsic viral communities.

### 3.4. Characterization of Key Pathogenic and Emerging Viruses

**Figure 4:**
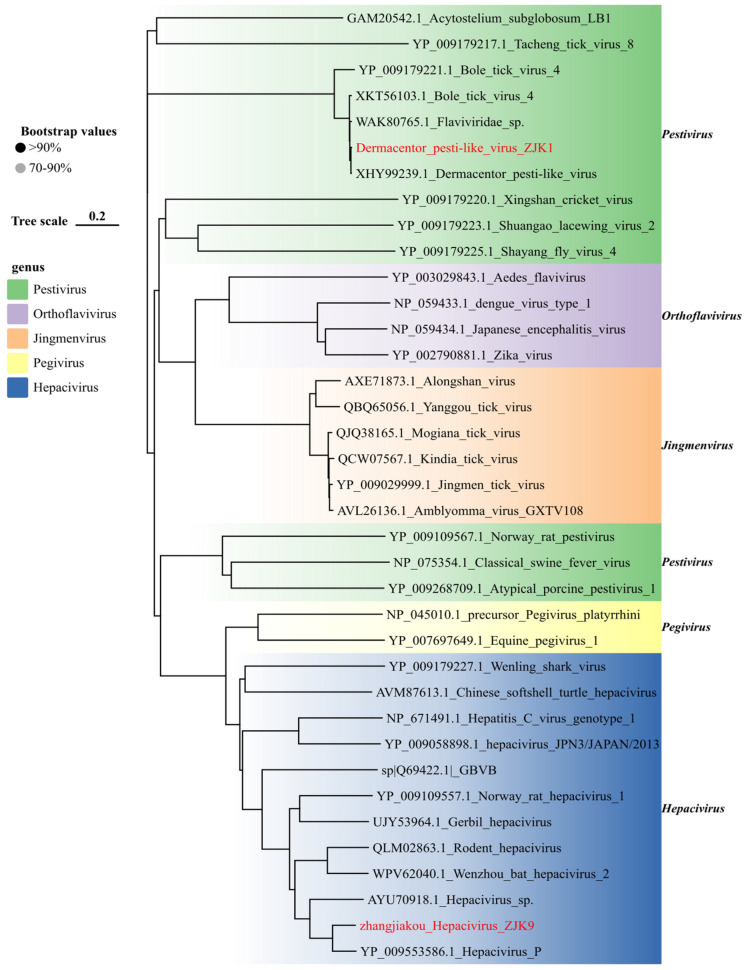
Phylogenetic analysis of *Flaviviridae* viral sequences identified in this study. Maximum-likelihood phylogenetic tree inferred from amino acid sequences of the RdRp region of representative members of the family *Flaviviridae*. The best-fit substitution model (LG + G + I) was selected using ModelFinder in IQ-TREE, and branch support was assessed with 1000 ultrafast bootstrap replicates. Sequences generated in this study are highlighted in red.

Phylogenetic analysis of the family *Flaviviridae* (Figure 4) revealed two divergent viral lineages circulating in Zhangjiakou tick populations. We initially identified a strain of Dermacentor pesti-like virus in *D. nuttalli*, which shared 98.8% nucleotide identity with reference sequences retrieved from the NCBI database. Given the evolutionary proximity of tick-associated pestiviruses to high-consequence veterinary pathogens, such as classical swine fever virus, the detection of this virus in local tick populations warrants further investigation regarding its host range and veterinary relevance. Furthermore, we characterized a putative novel agent in *H. japonica* ticks (collected from rodents), provisionally designated Zhangjiakou hepacivirus. Although BLAST analysis indicated 73.78% nucleotide identity to Hepacivirus P [33], phylogenetic reconstruction firmly positioned it within the Hepacivirus genus. To our knowledge, this constitutes the first description of this lineage in tick specimens, indicating that hepacivirus-related RNA can be recovered from tick-derived samples in this region, although its biological association with ticks remains unresolved. However, distinguishing between intrinsic vector competence and passive acquisition via the rodent blood meal remains critical; thus, further investigations including salivary gland screening and transmission assays are required to substantiate its status as a tick-borne pathogen.

**Figure 5:**
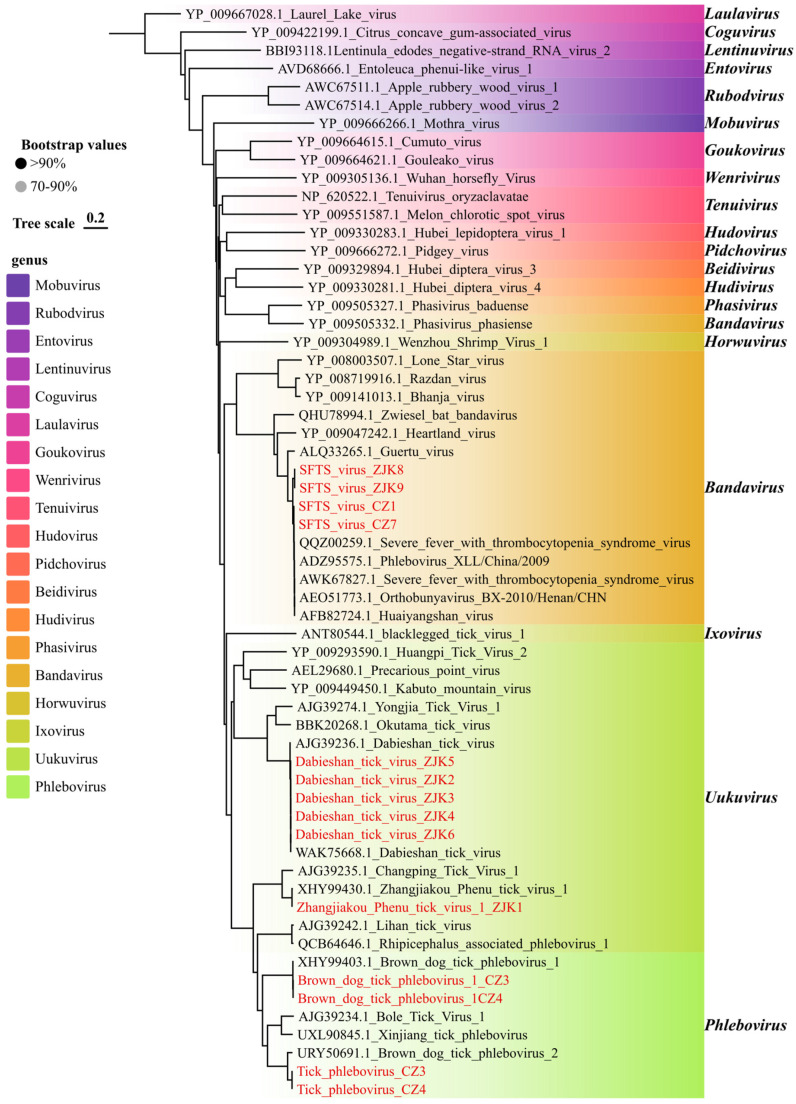
Phylogenetic analysis of *Phenuiviridae* viral sequences identified in this study. Maximum-likelihood phylogenetic tree inferred from amino acid sequences of the RdRp region and complete or near-complete genomic segments, as indicated in each panel. Trees were reconstructed in IQ-TREE using the best-fit substitution model (LG + G + I) selected by ModelFinder, and branch support was evaluated with 1000 ultrafast bootstrap replicates. Sequences generated in this study are highlighted in red.

The family *Phenuiviridae* emerged as the most significant group relevant to public health in this study [34], which has been identified in a growing number of mammalian taxa, including humans and livestock, via arthropod vectors [35]. This family was represented in our samples by five distinct viruses. Phylogenetic analysis of the RdRp domains confirmed the classification of these viruses into three established genera (Figure 5). Specifically, SFTSV clustered within the genus Bandavirus. Dabieshan tick virus and Zhangjiakou Phenu tick virus 1 were grouped within the genus Uukuvirus, while Brown dog tick phlebovirus 1 and Tick phlebovirus were classified under the genus Phlebovirus.

**Figure 6:**
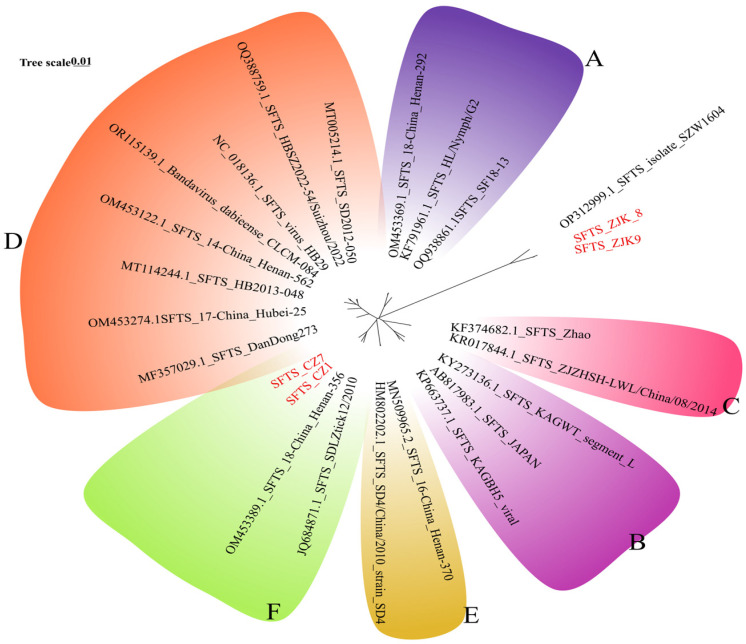
Maximum likelihood phylogenetic tree of the L segment nucleotide sequences of severe fever with thrombocytopenia syndrome virus (SFTSV). The tree was constructed using the general time-reversible (GTR) substitution model with gamma-distributed rate variation and a proportion of invariable sites (GTR + G + I). Bootstrap values (1000 replicates) are indicated at major nodes. The tree is rooted at the midpoint, and the six canonical SFTSV genotypes (**A**–**F**) are highlighted in distinct colored sectors. The SFTSV sequences identified in this study are marked in red and labeled with their sample identifiers. The scale bar represents the number of nucleotide substitutions per site.

Of greatest public health significance was the detection of SFTSV. Viral sequences were identified in four libraries (ZJK8, ZJK9, CZ1, and CZ7) prepared from *H. longicornis* and *D. nuttalli* collected in Zhangjiakou and Cangzhou (Figure 5). The virus was detected in 4/18 tick pools (22.2%) from Zhangjiakou and Cangzhou, underscoring the need for sustained SFTS surveillance in these areas. Notably, no SFTSV sequences were detected in any tick libraries from Shijiazhuang to date. We successfully assembled the complete L (6368 bp), M (3378 bp), and S (1744 bp) genomic segments, all of which retained intact open reading frames (ORFs). BLAST analysis against the NCBI database revealed high nucleotide identity (96.8–99.0%) with previously reported Chinese strains (Figure 6). These metatranscriptomic findings were subsequently validated by real-time RT-PCR. Phylogenetic reconstruction of the complete L, M, and S segments revealed a marked geographic stratification among the four SFTSV strains identified in this study (Figure 6, Figures S2 and S3). While the strains collected from Cangzhou (CZ1 and CZ7) consistently clustered within Genotype F across all three genomic segments, aligning with widespread lineages circulating in central and eastern China, the strains from Zhangjiakou (ZJK8 and ZJK9) exhibited a characteristic evolutionary trajectory. These Zhangjiakou strains formed a divergent lineage that did not cluster within any of the previously described canonical genotypes (A–F), instead grouping robustly with the SFTSV isolate SZW1604 from Inner Mongolia, sharing 96.8% nucleotide similarity with this closest relative. This phylogenetic pattern implies a specific epidemiological link between northern Hebei and the neighboring Inner Mongolia region, highlighting the co-circulation of both the predominant Genotype F and a unique lineage within Hebei Province. This genetic divergence suggests potential reassortment or the existence of a unique localized lineage. We additionally evaluated nucleotide diversity among the recovered SFTSV sequences, and the results are provided in the Supplementary Materials (Tables S6–S11).

Another significant finding was the detection of Dabieshan tick virus (DBSTV), a member of the family *Phenuiviridae* (Figure 5). DBSTV was highly prevalent in Zhangjiakou, being detected in 6/9 of tick pools from this region, but it was not detected in samples from the other two cities. This apparent geographic restriction suggests that local ecological conditions and tick population factors in Zhangjiakou may shape DBSTV maintenance and circulation, warranting further investigation. From Zhangjiakou samples, we recovered the complete L and S segments for five DBSTV strains. In contrast, the M segment was not detected in our RNA-seq data, consistent with previous reports describing the absence of M segments in related uukuviruses [36,37]. Phylogenetic analysis based on RdRp sequences showed that the DBSTV strains clustered closely with Yongjia tick virus 1 and Okutuma tick virus (Figure 5). Consistently, BLAST searches confirmed the high conservation of these viruses, showing that the DBSTV strains share up to 99.94% nucleotide identity with the closest reference sequences. We additionally evaluated nucleotide diversity among the recovered Dabieshan tick virus sequences, and the results are provided in the Supplementary Materials (Tables S12 and S13).

In the Cangzhou region, metagenomic sequencing of *H. longicornis* revealed the presence of Brown dog tick phlebovirus 1 and Tick phlebovirus (Figure 5). BLAST analysis revealed that these strains share 98.87% and 99.35% nucleotide identity with previously reported reference strains, respectively. Conversely, Zhangjiakou Phenu tick virus 1 was detected exclusively in a single library (ZJK1), showing 99.56% nucleotide identity to its closest known relative based on BLAST analysis.

### 3.5. Diversity of Additional Tick-Associated Viruses

**Figure 7:**
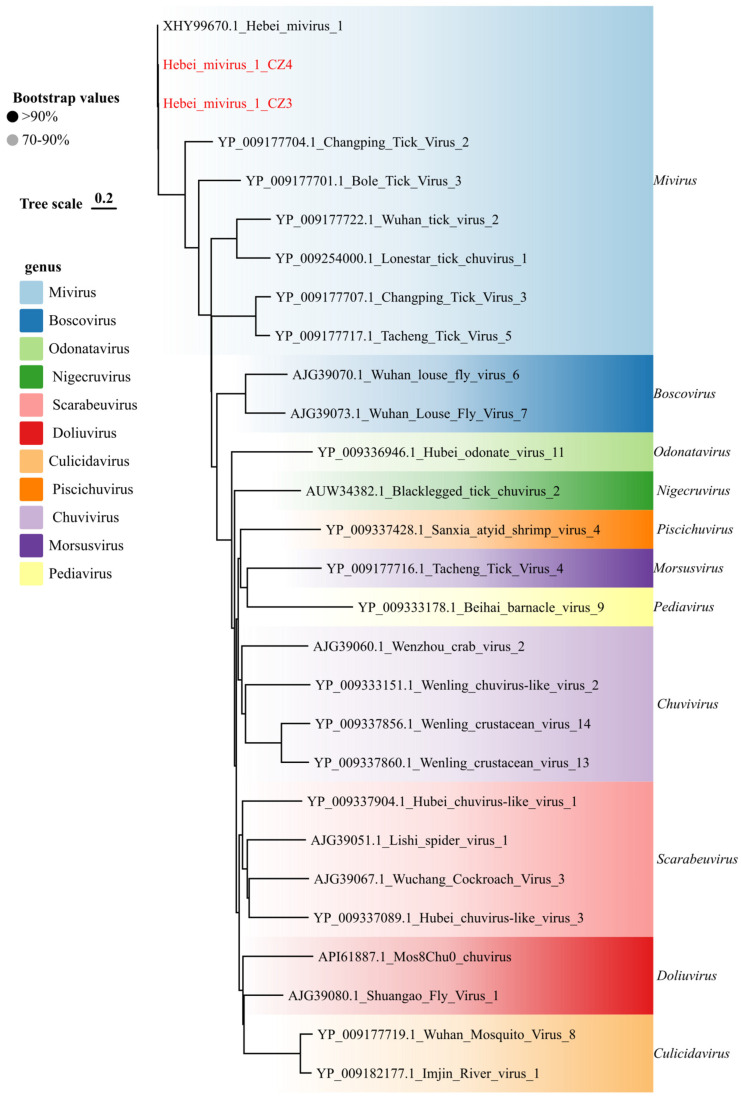
Phylogenetic analysis of *Chuviridae* viral sequences identified in this study. Maximum-likelihood phylogenetic tree inferred from amino acid sequences of the RdRp region and complete or near-complete genomic segments, as indicated in each panel. Trees were reconstructed in IQ-TREE using the best-fit substitution model (LG + G + I) selected by ModelFinder, and branch support was evaluated with 1000 ultrafast bootstrap replicates. Sequences generated in this study are highlighted in red.

We also characterized a diverse array of viruses belonging to families associated with arthropod vectors. Within the family *Chuviridae*, Hebei mivirus 1 was identified in two libraries from Cangzhou (CZ3 and CZ4). Phylogenetic reconstruction of the RdRp domain firmly placed these strains within the genus Mivirus (Figure 7), where they formed a well-supported monophyletic clade with a previously reported Chinese strain. This classification was further corroborated by whole-genome comparison, which revealed 96.4–99.9% nucleotide identity between our strains and existing isolates.

**Figure 8:**
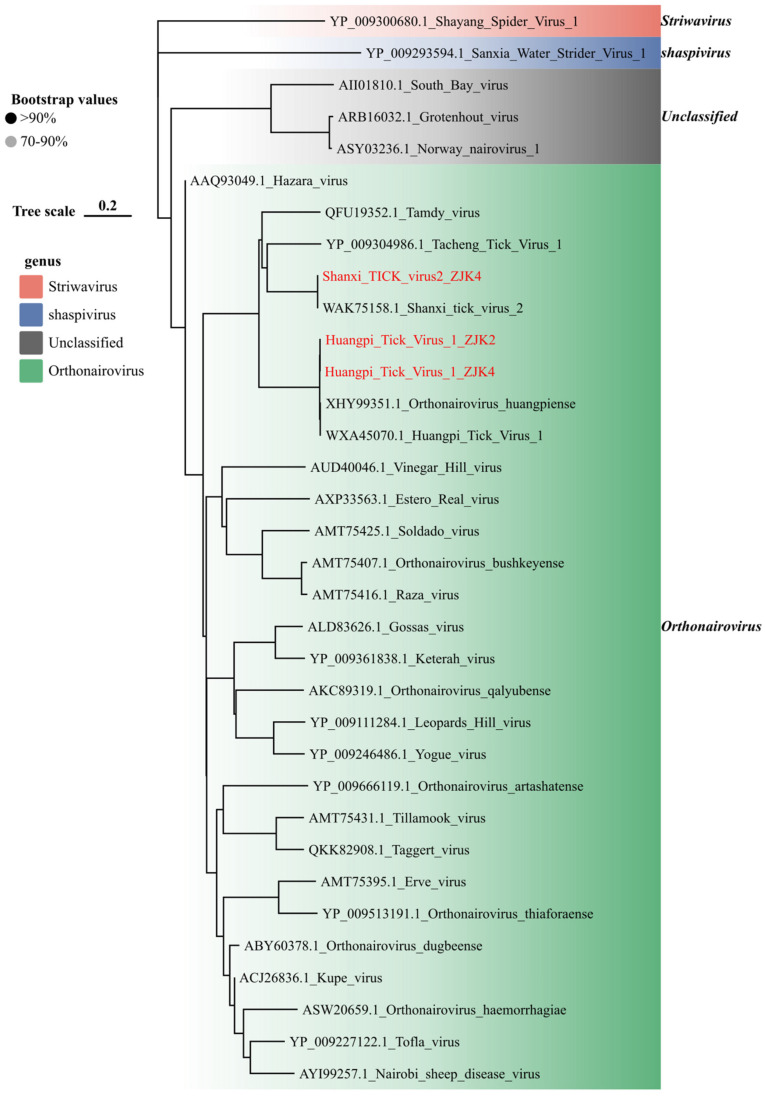
Phylogenetic analysis of *Nairoviridae* viral sequences identified in this study. Maximum-likelihood phylogenetic tree inferred from amino acid sequences of the RdRp region and complete or near-complete genomic segments, as indicated in each panel. Trees were reconstructed in IQ-TREE using the best-fit substitution model (LG + G + I) selected by ModelFinder, and branch support was evaluated with 1000 ultrafast bootstrap replicates. Sequences generated in this study are highlighted in red.

The family *Nairoviridae* was represented by two species detected in *H. longicornis* ticks from Zhangjiakou (Figure 8), both confirmed as members of the genus *Orthonairovirus* based on RdRp phylogeny. For Huangpi tick virus 1 (HTV-1), two strains were recovered, exhibiting high sequence conservation across the genome (S segment: 95.4–99.9%; M segment: 96.1–99.1%; L segment: 95.1–99.0%) relative to reference strains (Table S2). In contrast, Shanxi tick virus 2 (SXTV-2) was detected in a single library (ZJK4). BLAST analysis of this strain showed nucleotide similarities of 99.88%, 99.11%, and 99.42% for the L, M, and S segments, respectively, indicating a high degree of evolutionary stability with known isolates. This study is the first to systematically detect Huangpi tick virus 1 and Shanxi tick virus 2 in tick samples collected from the Zhangjiakou region of Hebei Province. Our findings expand the known geographical distribution of these two novel tick-borne nairoviruses in Hebei Province, fill the critical data gap in tick-borne nairovirus surveillance across the mountainous ecological region of northern Hebei, and simultaneously confirm that viruses belonging to the genus *Orthonairovirus* may maintain a stable local enzootic circulation within tick populations in the northern Hebei, China.

Our surveillance also identified members of the families *Rhabdoviridae* (Figure S4) and *Orthomyxoviridae* (Figure S5). In Zhangjiakou, Zhangjiakou rhabdo tick virus 2, a putative member of the genus Alphanemrhavirus, was detected in three *H. longicornis* pools, exhibiting 96.95% nucleotide identity to reference strains. From the same host species and region, we recovered Qingyang ortho tick virus 1. This strain showed high nucleotide similarity (98.85%) to known isolates and clustered phylogenetically with Yanbian ortho tick virus 1.

### 3.6. Detection of Plant- and Fungus-Associated Viruses

Beyond the putative tick-associated viruses described above, the metatranscriptomic data also contained viral sequences related to plant-, fungal-, or other non-vertebrate-associated viruses. These sequences may reflect environmental contamination, fungal associations, residual material on the tick surface, or incidental exposure rather than active infection of ticks. Notably, we recovered 12 viral sequences exhibiting >90% nucleotide similarity to strains previously documented in ticks, suggesting a stable core virome across the region. Taxonomically, these sequences fall into five families, with five belonging to *Botourmiaviridae*, three to *Tombusviridae*, two to *Totiviridae*, one to *Parvoviridae* and one to *Solemoviridae* (Figures S6–S9). Ecologically, this diversity points to distinct biological sources. The *Parvoviridae* and *Botourmiaviridae* are likely associated with tick-specific infections or fungal symbionts, whereas the *Tombusviridae* and *Solemoviridae* which are known plant viruses may derive from the host’s environmental exposure or dietary ingestion [38]. The identification of these diverse viral signatures, which share high homology with regional reference strains, provides contextual support for the plausibility of our metatranscriptomic detections.

## 4. Discussion

The expanding geographic range of tick populations and increasing human activities in tick-endemic habitats have heightened concerns regarding the risk of tick-borne viral spillover [39,40,41]. In this study, we conducted a systematic metatranscriptomic investigation of tick viromes across three ecologically distinct regions of Hebei Province, a transitional zone characterized by diverse topography ranging from the Bashang Plateau and Taihang Mountains in the northwest to the coastal plains of the Bohai Sea in the east. Notably, these regions exhibit marked variations in topography, altitude, and vegetation features, which may exert a profound impact on tick distribution and viral community assembly [42]. From 986 ticks, we identified 25 RNA viruses spanning 12 families, filling a critical knowledge gap regarding viral diversity in North China. These findings suggest that the detected viral RNA profiles varied across the sampled tick groups and collection contexts. However, these patterns should be interpreted cautiously given the pooled design and the absence of directly measured ecological variables. Crucially, the detection of *H. asiaticum* in Hebei represents an expansion of its documented regional distribution. Traditionally restricted to the arid Northwest, Its detection in Hebei may reflect range expansion, livestock-associated introduction, or previous underdetection; dedicated ecological surveillance will be required to distinguish among these possibilities. As *H. asiaticum* is the primary biological vector for CCHFV, its presence creates a silent receptive environment for this virulent pathogen [43,44]. Although our targeted screening for CCHFV yielded negative results, the establishment of its competent vector represents a potential risk factor for future CCHFV transmission should the virus be introduced into the region.

We identified several divergent viral clades, broadening the known tick virome. Notably, the Zhangjiakou Hepacivirus, detected in *H. japonica*, shares <80% nucleotide identity with hepacivirus P and meets the ICTV criteria for a new subtype. Hepacivirus P was originally identified in long-tailed ground squirrels in Xinjiang, northwestern China [33]. Our detection of a closely related strain in Hebei, thousands of kilometers eastward, implies widespread circulation of this viral lineage across the rodent populations of northern China. This finding extends the known phylogenetic and ecological breadth of the genus, which has been identified in a growing number of mammalian taxa, including cattle, rodents, bats, and primates [45,46,47,48,49].

To our knowledge, this constitutes the first report of a hepacivirus sequence within tick specimens, raising intriguing questions about transmission ecology. Historically, hepaciviruses are presumed to be vertebrate-specific, with no known arthropod vectors. The association of Zhangjiakou Hepacivirus with *H. japonica* suggests that ticks could theoretically serve as mechanical vectors or paratenic hosts, passively transferring the virus between rodent reservoirs and other susceptible hosts. However, biological plausibility necessitates caution. Hepaciviruses, including HCV, are strictly hepatotropic and typically rely on the liver-specific microRNA-122 (miR-122) for viral RNA stability and replication [48,50,51]. Since ticks lack a liver and presumably the specific miR-122 machinery, active replication within the vector seems unlikely unless this novel lineage has evolved alternative replication mechanisms [52]. Given that the ticks were collected directly from rodents, the most parsimonious explanation is “passive acquisition” via a viremic blood meal.

At present, there is no evidence that any hepacivirus is truly “tick-borne” in the biological sense. Discriminating between mechanical carriage and bona fide vector competence will require targeted studies. From a public health perspective, the repeated emergence of highly divergent hepaciviruses in synanthropic rodents highlights a potential source of cross-species spillover [53,54,55]. Our findings underscore the value of integrating vector and wildlife sampling within a One Health framework. Future work in Zhangjiakou should include systematic screening of small mammals and exploratory serological surveys in high-risk human populations.

The detection of SFTSV in 22.2% of tick pools from Zhangjiakou and Cangzhou has substantial public health implications and poses serious challenges for disease prevention and control in Hebei Province and across northern China. As a high-consequence tick-borne pathogen associated with severe and sometimes fatal human infection [56], this finding not only fills a critical surveillance gap for SFTSV vectors in Hebei but also strongly supports local circulation of SFTSV in the surveyed areas. Notably, the involved tick species include *H. longicornis* and *D. nuttalli*, which are dominant local species with broad contact rates with humans and animals [7]. The relatively high positivity rate of 22.2% indicates detectable circulation of SFTSV among sampled tick pools and suggests a potential risk of human exposure, particularly among outdoor workers and agricultural communities with frequent contact with tick habitats [57].

While SFTSV has not yet been detected in tick libraries from Shijiazhuang, the results from Zhangjiakou and Cangzhou clearly highlight a geographical disparity in SFTSV prevalence within Hebei Province. Consequently, public health interventions must be more geographically targeted. In Zhangjiakou and Cangzhou, immediate actions should include enhanced surveillance for acute febrile cases, improved early diagnosis and reporting capabilities for SFTS, and targeted health education campaigns to raise awareness about tick bite prevention among at risk groups [58]. Additionally, sustained monitoring of SFTSV prevalence in local tick vectors and animal reservoirs is crucial for tracking the dynamic transmission of the virus and informing timely adjustments to prevention and control strategies [59]. Furthermore, to ensure comprehensive provincial public health security, even in areas where SFTSV has not currently been identified, such as Shijiazhuang, it is imperative to maintain a high level of vigilance, strengthen baseline surveillance, and conduct ongoing risk assessments to preempt potential viral spread or the emergence of new foci. Collectively, these findings position SFTSV as a priority tick-borne pathogen for routine surveillance across Hebei Province, with a specific focus on the identified high-risk regions of Zhangjiakou and Cangzhou.

Perhaps the most significant public health implication is the discovery of the geographic stratification of SFTSV genotypes within Hebei. We identified two characteristic evolutionary lineages circulating in spatially separated niches. The Cangzhou strains clustered with Genotype F [60], the predominant lineage in central and eastern China [61]. In sharp contrast, the Zhangjiakou strains formed a deeply divergent Northern Lineage that clustered robustly with the SZW1604 strain from Inner Mongolia [62]. This implies that Hebei sits at a critical ecological intersection, the Zhangjiakou lineage likely represents a spillover from the Inner Mongolian steppe ecosystem, facilitated by the contiguous landscape. The co-circulation of these divergent genotypes warrants urgent surveillance, as reassortment between lineages could potentially give rise to variants with altered pathogenicity. These findings underscore the need for continuous genomic surveillance to evaluate public-health risk and vaccine relevance.

Beyond the novel hepacivirus and SFTSV, our study highlights the circulation of several neglected viruses with pathogenic potential. Specifically, the detection of Shanxi tick virus 2 [63] and Huangpi tick virus 1 [64] (*Nairoviridae*) warrants vigilance. Both belong to the genus *Orthonairovirus* [65], which includes the lethal CCHFV. While frequently detected, their phylogenetic proximity to human pathogens suggests their risk should not be underestimated. Similarly, Dabieshan tick virus (DBSTV) emerged as a dominant virus in Zhangjiakou (66.67% poor positivity). Given that uukuviruses can cause febrile illness, the high burden of DBSTV suggests a robust enzootic cycle, raising the possibility of spillover [66]. We strongly advocate for shifting surveillance strategies to a multi-target approach, routinely screening for these neglected viruses in febrile patients and livestock. Additionally, we identified a strain of Dermacentor pesti-like virus in *D. nuttalli*. Given the close relationship of pestiviruses to livestock pathogens like Classical Swine Fever Virus, its presence in a livestock-dense region requires veterinary vigilance [67].

Taken together, our findings describe heterogeneity in viral RNA profiles across sampled tick groups in Hebei Province. In the southern alluvial plain, the viral community was relatively concentrated, with the F-type lineage of SFTSV as the dominant taxon. In contrast, the northern Bashang grassland exhibited greater viral diversity, harboring not only SFTSV but also Dabieshan tick virus, a novel hepacivirus sequence, and multiple members of the genus *Orthonairovirus*. Furthermore, it indicates that tick-borne viruses in the transitional zone between the mountainous areas of northern Hebei and the Inner Mongolian Plateau may have evolutionary origins and geographic transmission characteristics different from those of strains circulating in the North China Plain. These findings provide new baseline data for understanding the genetic diversity, geographic distribution patterns, and risk surveillance of tick-borne viruses in northern China.

Furthermore, our analysis revealed a characteristic community of plant and insect viruses [68]. These viruses most likely represent surface contamination acquired during contact with soil and vegetation while questing. Ticks are obligate hematophagous arthropods and do not ingest plant material; therefore, the detection of plant-associated viruses cannot be attributed to dietary ingestion of herbivorous vertebrate hosts. Rather, physical contact with plant surfaces during the host-seeking process provides a plausible route for the passive acquisition of plant viral RNA on the tick’s exterior, which would then be co-extracted during whole-tick homogenization [69,70].

A major limitation of this study is the use of large and uneven pool sizes. Although pooling enabled broad exploratory screening across a substantial number of ticks, it precludes estimation of individual-level infection prevalence. Additionally, the pathogenicity of novel viruses remains to be verified. Because engorged ticks were included in the dataset, some differences among regional groups may reflect variation in vertebrate host exposure rather than true differences in intrinsic tick-associated viromes. Another limitation is that no negative controls were included in the shotgun metatranscriptomic workflow. Therefore, low-abundance environmental, laboratory, or reagent-derived contamination cannot be fully excluded. These limitations will be a key focus of our future research.

## 5. Conclusions

In summary, this study presents a comprehensive characterization of the tick-borne RNA virome in Hebei Province, identifying both established pathogens and previously unrecognized viral lineages in tick-derived samples from Hebei Province. These results provide critical baseline data for elucidating the viral diversity and genetic relationships of tick-borne viruses in northern China. Strengthening continuous surveillance and re-examining archived tick specimens with high-throughput sequencing will be vital for long-term monitoring of these viruses. Sustained, region-specific monitoring will enhance our understanding of tick virus co-evolution and offer scientific guidance for the early warning, prevention, and control of emerging tick-borne diseases. Collectively, these findings establish a foundation for an integrated One Health surveillance framework, emphasizing the need for targeted monitoring across both the lowland plains and the northern mountainous corridors of Hebei.

## Data Availability

All the data generated during the current study are included in the manuscript. All sequencing reads have been deposited in the CNCB databases (www.cncb.ac.cn) under the GSA accession CRA036952. Relevant virus genome sequences have been deposited in the GenBase databases (accession: C_AA136341.1 to C_AA136393.1). Additional data related to this article may be requested from the corresponding authors.

## Supplementary Materials

The following supporting information can be downloaded at: https://www.mdpi.com/article/10.3390/v18040443/s1, Table S1: Distribution of tick collection areas in Hebei Province, China; Table S2: Viruses identified from each library in the present study; Table S3: Sequence information; Table S4: Details of sequencing data; Table S5: Library-level read counts of detected viral taxa across pooled tick libraries; Table S6: SFTS-L fragment nucleotide variation sites; Table S7: SFTS-L fragment nucleotide similarity; Table S8: SFTS-M fragment nucleotide variation sites; Table S9: SFTS-M fragment nucleotide similarity; Table S10: SFTS-S fragment nucleotide variation sites; Table S11: SFTS-S fragment nucleotide similarity; Table S12: DBTV nucleotide variation sites; Table S13: DBTV nucleotide similarity. Figure S1: Library-level summaries of sequencing composition and alpha diversity across pooled tick libraries; Figure S2: Maximum likelihood phylogenetic tree of the M segment nucleotide sequences of severe fever with thrombocytopenia syndrome virus (SFTSV); Figure S3: Maximum likelihood phylogenetic tree of the S segment nucleotide sequences of severe fever with thrombocytopenia syndrome virus (SFTSV); Figure S4: Phylogenetic analysis of *Rhabdoviridae* viral sequences identified in this study; Figure S5: Phylogenetic analysis of *Orthomyxoviridae* viral sequences identified in this study; Figure S6: Phylogenetic analysis of *Parvoviridae* viral sequences identified in this study; Figure S7: Phylogenetic analysis of *Tombusviridae* viral sequences identified in this study; Figure S8: Phylogenetic analysis of *Totiviridae* viral sequences identified in this study; Figure S9: Phylogenetic analysis of *Botourmiaviridae* viral sequences identified in this study.

## References

[1] Zhang Y.-K., Zhang X.-Y., Liu J.-Z. (2019). Ticks (Acari: Ixodoidea) in China: Geographical distribution, host diversity, and specificity. Arch. Insect Biochem. Physiol..

[2] Zhou H., Xu L., Shi W. (2023). The human-infection potential of emerging tick-borne viruses is a global public health concern. Nat. Rev. Microbiol..

[3] Moming A., Bai Y., Wang J., Zhang Y., Tang S., Fan Z., Deng F., Shen S. (2024). The Known and Unknown of Global Tick-Borne Viruses. Viruses.

[4] Hawman D.W., Feldmann H. (2023). Crimean-Congo haemorrhagic fever virus. Nat. Rev. Microbiol..

[5] Lindquist L., Vapalahti O. (2008). Tick-borne encephalitis. Lancet.

[6] Yu X.J., Liang M.F., Zhang S.Y., Liu Y., Li J.D., Sun Y.L., Zhang L., Zhang Q.F., Popov V.L., Li C. (2011). Fever with thrombocytopenia associated with a novel bunyavirus in China. N. Engl. J. Med..

[7] Zhao L., Li J., Cui X., Jia N., Wei J., Xia L., Wang H., Zhou Y., Wang Q., Liu X. (2020). Distribution of Haemaphysalis longicornis and associated pathogens: Analysis of pooled data from a China field survey and global published data. Lancet Planet. Health.

[8] Wang Z.-D., Wang B., Wei F., Han S.-Z., Zhang L., Yang Z.-T., Yan Y., Lv X.-L., Li L., Wang S.-C. (2019). A New Segmented Virus Associated with Human Febrile Illness in China. N. Engl. J. Med..

[9] Ji N., Wang N., Liu G., Zhao S., Liu Z., Tan W., Wang S., Sheng J., Li F., Wang Y. (2023). Tacheng Tick Virus 1 and Songling Virus Infection in Great Gerbils (*Rhombomys opimus*) in Northwestern China. J. Wildl. Dis..

[10] Zhang M.-Z., Bian C., Ye R.-Z., Cui X.-M., Chu Y.-L., Yao N.-N., Xu X.-W., Ye J.-L., Chen L., Yang J.-H. (2025). Human Infection with a Novel Tickborne Orthonairovirus Species in China. N. Engl. J. Med..

[11] Zhang X.-A., Ma Y.-D., Zhang Y.-F., Hu Z.-Y., Zhang J.-T., Han S., Wang G., Li S., Wang X., Tang F. (2024). A New Orthonairovirus Associated with Human Febrile Illness. N. Engl. J. Med..

[12] Wang R., Liu S., Sun H., Xu C., Wen Y., Wu X., Zhang W., Nie K., Li F., Fu S. (2023). Metatranscriptomics Reveals the RNA Virome of Ixodes Persulcatus in the China-North Korea Border, 2017. Viruses.

[13] Ni X.-B., Cui X.-M., Liu J.-Y., Ye R.-Z., Wu Y.-Q., Jiang J.-F., Sun Y., Wang Q., Shum M.H.-H., Chang Q.-C. (2023). Metavirome of 31 tick species provides a compendium of 1,801 RNA virus genomes. Nat. Microbiol..

[14] Wingett S.W., Andrews S. (2018). FastQ Screen: A tool for multi-genome mapping and quality control. F1000Research.

[15] Bolger A.M., Lohse M., Usadel B. (2014). Trimmomatic: A flexible trimmer for Illumina sequence data. Bioinformatics.

[16] Li D., Liu C.-M., Luo R., Sadakane K., Lam T.-W. (2015). MEGAHIT: An ultra-fast single-node solution for large and complex metagenomics assembly via succinct de Bruijn graph. Bioinformatics.

[17] Gurevich A., Saveliev V., Vyahhi N., Tesler G. (2013). QUAST: Quality assessment tool for genome assemblies. Bioinformatics.

[18] Buchfink B., Reuter K., Drost H.-G. (2021). Sensitive protein alignments at tree-of-life scale using DIAMOND. Nat. Methods.

[19] Huson D.H., Auch A.F., Qi J., Schuster S.C. (2007). MEGAN analysis of metagenomic data. Genome Res..

[20] Camacho C., Coulouris G., Avagyan V., Ma N., Papadopoulos J., Bealer K., Madden T.L. (2009). BLAST+: Architecture and applications. BMC Bioinform..

[21] Langmead B., Salzberg S.L. (2012). Fast gapped-read alignment with Bowtie 2. Nat. Methods.

[22] Zhao Y., Li M.-C., Konaté M.M., Chen L., Das B., Karlovich C., Williams P.M., Evrard Y.A., Doroshow J.H., McShane L.M. (2021). TPM, FPKM, or Normalized Counts? A Comparative Study of Quantification Measures for the Analysis of RNA-seq Data from the NCI Patient-Derived Models Repository. J. Transl. Med..

[23] Pettersson J.H.O., Ellström P., Ling J., Nilsson I., Bergström S., González-Acuña D., Olsen B., Holmes E.C. (2020). Circumpolar diversification of the Ixodes uriae tick virome. PLoS Pathog..

[24] Plowright R.K., Peel A.J., Streicker D.G., Gilbert A.T., McCallum H., Wood J., Baker M.L., Restif O. (2016). Transmission or Within-Host Dynamics Driving Pulses of Zoonotic Viruses in Reservoir-Host Populations. PLoS Neglected Trop. Dis..

[25] Katoh K., Standley D.M. (2013). MAFFT multiple sequence alignment software version 7: Improvements in performance and usability. Mol. Biol. Evol..

[26] Capella-Gutiérrez S., Silla-Martínez J.M., Gabaldón T. (2009). trimAl: A tool for automated alignment trimming in large-scale phylogenetic analyses. Bioinformatics.

[27] Minh B.Q., Schmidt H.A., Chernomor O., Schrempf D., Woodhams M.D., von Haeseler A., Lanfear R. (2020). IQ-TREE 2: New Models and Efficient Methods for Phylogenetic Inference in the Genomic Era. Mol. Biol. Evol..

[28] Kalyaanamoorthy S., Minh B.Q., Wong T.K.F., von Haeseler A., Jermiin L.S. (2017). ModelFinder: Fast model selection for accurate phylogenetic estimates. Nat. Methods.

[29] Tamura K., Stecher G., Kumar S. (2021). MEGA11: Molecular Evolutionary Genetics Analysis Version 11. Mol. Biol. Evol..

[30] Liu C., Cui Y., Li X., Yao M. (2021). microeco: An R package for data mining in microbial community ecology. FEMS Microbiol. Ecol..

[31] Bayindir Gümüş A., Keser A., Gökgöz M., Güngüneş A. (2024). Glycaemic index and glycaemic load of selected packaged vegan foods. Nutr. Bull..

[32] Ding W., Goldberg D., Zhou W. (2023). PyComplexHeatmap: A Python package to visualize multimodal genomics data. Imeta.

[33] Li L.-L., Liu M.-M., Shen S., Zhang Y.-J., Xu Y.-L., Deng H.-Y., Deng F., Duan Z.-J. (2019). Detection and characterization of a novel hepacivirus in long-tailed ground squirrels (Spermophilus undulatus) in China. Arch. Virol..

[34] Sasaya T., Palacios G., Briese T., Di Serio F., Groschup M.H., Neriya Y., Song J.-W., Tomitaka Y. (2023). ICTV Virus Taxonomy Profile: Phenuiviridae 2023. J. Gen. Virol..

[35] Sun M.-H., Ji Y.-F., Li G.-H., Shao J.-W., Chen R.-X., Gong H.-Y., Chen S.-Y., Chen J.-M. (2022). Highly adaptive Phenuiviridae with biomedical importance in multiple fields. J. Med. Virol..

[36] Mekata H., Kobayashi I., Okabayashi T. (2023). Detection and phylogenetic analysis of Dabieshan tick virus and Okutama tick virus in ticks collected from Cape Toi, Japan. Ticks Tick-Borne Dis..

[37] Ma C., Zhang R., Zhou H., Yu G., Yu L., Li J., Cui M., Carr M.J., Zhang Z., Shi W. (2022). Prevalence and genetic diversity of Dabieshan tick virus in Shandong Province, China. J. Infect..

[38] Sõmera M., Fargette D., Hébrard E., Sarmiento C., Ictv Report C. (2021). ICTV Virus Taxonomy Profile: Solemoviridae 2021. J. Gen. Virol..

[39] Gabriel A.N.A., Wang X.-Y., Fornah L., Belete A.G., Russo M.T., Lota L.K., Mekonnen T.D., Shimbre M.S. (2025). Tick Diversity, Emerging Tick-Borne Pathogens, and Public Health Implications Across Africa: A Systematic Review. Acta Parasitol..

[40] Kamau M., Ergunay K., Bourke B.P., Mutura J., Lebunge R., Ochieng G., Gathii K., Waitumbi J., Mutai B., Hassell J. (2025). Potential spillover investigated by metagenome sequencing in Laikipia, Kenya reveals tick-borne pathogens and a novel bunyavirus. One Health.

[41] Vandegrift K.J., Kapoor A. (2019). The Ecology of New Constituents of the Tick Virome and Their Relevance to Public Health. Viruses.

[42] Tian D., Ye R.-Z., Li Y.-Y., Wang N., Gao W.-Y., Wang B.-H., Lin Z.-T., Zhu W.-J., Wang Q.-S., Liu Y.-T. (2025). Virome specific to tick genus with distinct ecogeographical distribution. Microbiome.

[43] Telmadarraiy Z., Chinikar S., Vatandoost H., Faghihi F., Hosseini-Chegeni A. (2015). Vectors of Crimean Congo Hemorrhagic Fever Virus in Iran. J. Arthropod-Borne Dis..

[44] Choubdar N., Oshaghi M.A., Rafinejad J., Pourmand M.R., Maleki-Ravasan N., Salehi-Vaziri M., Telmadarraiy Z., Karimian F., Koosha M., Rahimi-Foroushani A. (2019). Effect of Meteorological Factors on Hyalomma Species Composition and Their Host Preference, Seasonal Prevalence and Infection Status to Crimean-Congo Haemorrhagic Fever in Iran. J. Arthropod-Borne Dis..

[45] Chen J.-T., Chen K.-J., Wu K.-W., Yi S.-H., Shao J.-W. (2024). Identification and epidemiology of a novel Hepacivirus in domestic ducks in Hunan province, China. Front. Vet. Sci..

[46] Quan P.-L., Firth C., Conte J.M., Williams S.H., Zambrana-Torrelio C.M., Anthony S.J., Ellison J.A., Gilbert A.T., Kuzmin I.V., Niezgoda M. (2013). Bats are a major natural reservoir for hepaciviruses and pegiviruses. Proc. Natl. Acad. Sci. USA.

[47] Corman V.M., Grundhoff A., Baechlein C., Fischer N., Gmyl A., Wollny R., Dei D., Ritz D., Binger T., Adankwah E. (2015). Highly divergent hepaciviruses from African cattle. J. Virol..

[48] Pfaender S., Brown R.J., Pietschmann T., Steinmann E. (2014). Natural reservoirs for homologs of hepatitis C virus. Emerg. Microbes Infect..

[49] Drexler J.F., Corman V.M., Müller M.A., Lukashev A.N., Gmyl A., Coutard B., Adam A., Ritz D., Leijten L.M., van Riel D. (2013). Evidence for novel hepaciviruses in rodents. PLoS Pathog..

[50] Jopling C. (2012). Liver-specific microRNA-122: Biogenesis and function. RNA Biol..

[51] Filipowicz W., Grosshans H. (2011). The liver-specific microRNA miR-122: Biology and therapeutic potential. Epigenetics and Disease.

[52] Bandiera S., Pfeffer S., Baumert T.F., Zeisel M.B. (2014). miR-122—A key factor and therapeutic target in liver disease. J. Hepatol..

[53] Borremans B., Faust C., Manlove K.R., Sokolow S.H., Lloyd-Smith J.O. (2019). Cross-species pathogen spillover across ecosystem boundaries: Mechanisms and theory. Philos. Trans. R. Soc. Lond. B Biol. Sci..

[54] Carlson C.J., Albery G.F., Merow C., Trisos C.H., Zipfel C.M., Eskew E.A., Olival K.J., Ross N., Bansal S. (2022). Climate change increases cross-species viral transmission risk. Nature.

[55] Zhang X.-L., Yao X.-Y., Zhang Y.-Q., Lv Z.-H., Liu H., Sun J., Shao J.-W. (2022). A Highly Divergent Hepacivirus Identified in Domestic Ducks Further Reveals the Genetic Diversity of Hepaciviruses. Viruses.

[56] Casel M.A., Park S.J., Choi Y.K. (2021). Severe fever with thrombocytopenia syndrome virus: Emerging novel phlebovirus and their control strategy. Exp. Mol. Med..

[57] Cassens J., Larson S., Keller K., Alexander B.H., Bender J.B., Oliver J.D. (2025). Estimating Infected Blacklegged Tick Encounters Among Outdoor Workers in Minnesota. EcoHealth.

[58] Rao A.S., Pai B.H.K., Adithi K., Shenoy R., Nayak S. (2025). Comprehensive review and early detection strategies for severe fever with thrombocytopenia: Insights from epidemiology, diagnostics, and evolving research with machine learning. Virusdisease.

[59] Lu H.-Y., Wu G.-D., Peng M., Wu L.-B., Luo Y.-M., Xia B., Xiong D., Qin X.-R., Guo F., Yu X.-J. (2025). SFTSV Prevalence in Ticks and Livestock in an SFTSV-Endemic Area in Central China. Pathogens.

[60] Cai Y., Wei Y., Li L., Geng M., Zheng Y., Zhang X., Han Z., Zhang Y., Xu Y., Han X. (2025). Identification of Endemic Region for Severe Fever with Thrombocytopenia Syndrome in an Alluvial Plain of Hebei Province, China. Viruses.

[61] Sheng R., Cheng T., Wang Y., Wen H. (2025). Molecular evolution and geographic migration of severe fever with thrombocytopenia syndrome virus in Asia. PLoS Pathog..

[62] Kong Y., Zhang G., Jiang L., Wang P., Zhang S., Zheng X., Li Y. (2022). Metatranscriptomics Reveals the Diversity of the Tick Virome in Northwest China. Microbiol. Spectr..

[63] Li F., Wang R., Zhang B., Jin G., Song J., Gou W., Sun C., Yin Q., Li D., Nie K. (2025). Neonatal mouse model reveals pathogenesis of Shanxi Tick Virus 2 isolated from Haemaphysalis longicornis. Virol. J..

[64] Kuhn J.H., Wiley M.R., Rodriguez S.E., Bào Y., Prieto K., Travassos da Rosa A.P.A., Guzman H., Savji N., Ladner J.T., Tesh R.B. (2016). Genomic Characterization of the Genus Nairovirus (Family Bunyaviridae). Viruses.

[65] Ariizumi T., Tabata K., Itakura Y., Kobayashi H., Hall W.W., Sasaki M., Sawa H., Matsuno K., Orba Y. (2024). Establishment of a lethal mouse model of emerging tick-borne orthonairovirus infections. PLoS Pathog..

[66] Han X., Gao S., Xin Q., Yang M., Bi Y., Jiang F., Zeng Z., Kan W., Wang T., Chen Q. (2024). Spatial risk of Haemaphysalis longicornis borne Dabieshan tick virus (DBTV) in China. J. Med. Virol..

[67] Beer M., Goller K.V., Staubach C., Blome S. (2015). Genetic variability and distribution of Classical swine fever virus. Anim. Health Res. Rev..

[68] Jeger M.J. (2020). The Epidemiology of Plant Virus Disease: Towards a New Synthesis. Plants.

[69] Geoghegan J.L., Senior A.M., Holmes E.C. (2016). Pathogen population bottlenecks and adaptive landscapes: Overcoming the barriers to disease emergence. Proc. Biol. Sci..

[70] Geoghegan J.L., Duchêne S., Holmes E.C. (2017). Comparative analysis estimates the relative frequencies of co-divergence and cross-species transmission within viral families. PLoS Pathog..

