# Supplementary material for: Metatranscriptomic Analysis of Tick Virome Diversity in Hebei Province, China
Source: Viruses. 2026 Apr 7;18(4):443. doi: 10.3390/v18040443 (PMC13120621; doi:10.3390/v18040443)
Supplement: Supplementary file 1 [file viruses-18-00443-s001.zip › Figure S5.pdf]

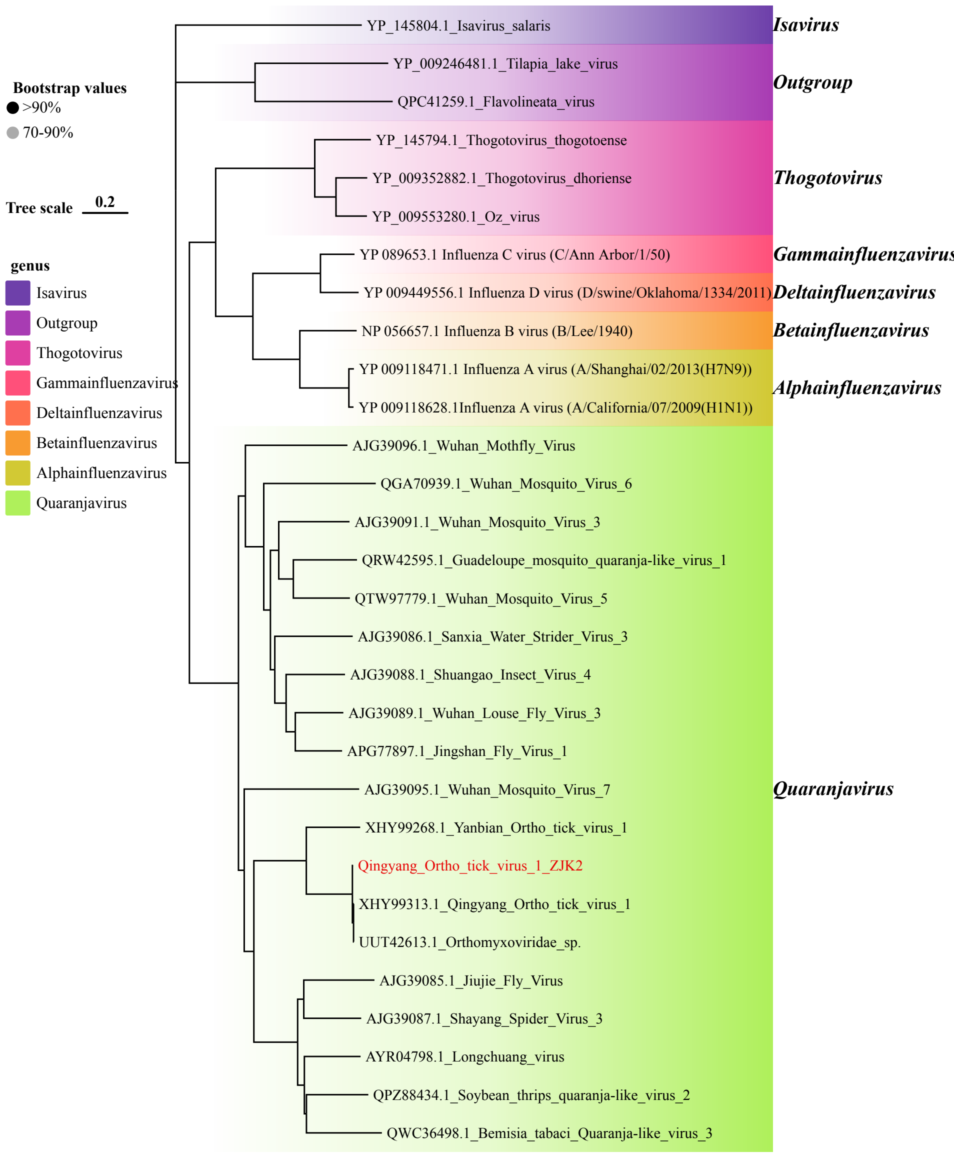
Bootstrap values
>90%
70-90%
Tree scale 0.2
genus
Isavirus
Outgroup
Thogotovirus
Gammainfluenzavirus
Deltainfluenzavirus
Betainfluenzavirus
Alphainfluenzavirus
Quaranjavirus
YP_145804.1_Isavirus_salaris
YP_009246481.1_Tilapia_lake_virus
QPC41259.1_Flavolineata_virus
YP_145794.1_Thogotovirus_thogotoense
YP_009352882.1_Thogotovirus_dhoriense
YP_009553280.1_Oz_virus
YP 089653.1 Influenza C virus (C/Ann Arbor/1/50)
YP 009449556.1 Influenza D virus (D/swine/Oklahoma/1334/2011)
NP 056657.1 Influenza B virus (B/Lee/1940)
YP 009118471.1 Influenza A virus (A/Shanghai/02/2013(H7N9))
YP 009118628.1Influenza A virus (A/California/07/2009(H1N1))
AJG39096.1_Wuhan_Mothfly_Virus
QGA70939.1_Wuhan_Mosquito_Virus_6
AJG39091.1_Wuhan_Mosquito_Virus_3
QRW42595.1_Guadeloupe_mosquito_quaranja-like_virus_1
QTW97779.1_Wuhan_Mosquito_Virus_5
AJG39086.1_Sanxia_Water_Strider_Virus_3
AJG39088.1_Shuangao_Insect_Virus_4
AJG39089.1_Wuhan_Louse_Fly_Virus_3
APG77897.1_Jingshan_Fly_Virus_1
AJG39095.1_Wuhan_Mosquito_Virus_7
XHY99268.1_Yanbian_Ortho_tick_virus_1
Qingyang_Ortho_tick_virus_1_ZJK2
XHY99313.1_Qingyang_Ortho_tick_virus_1
UUT42613.1_Orthomyxoviridae_sp.
AJG39085.1_Jiujie_Fly_Virus
AJG39087.1_Shayang_Spider_Virus_3
AYR04798.1_Longchuang_virus
QPZ88434.1_Soybean_thrips_quaranja-like_virus_2
QWC36498.1_Bemisia_tabaci_Quaranja-like_virus_3
Isavirus
Outgroup
Thogotovirus
Gammainfluenzavirus
Deltainfluenzavirus
Betainfluenzavirus
Alphainfluenzavirus
Quaranjavirus
