# Supplementary figures and images for: Metatranscriptomic Analysis of Tick Virome Diversity in Hebei Province, China
Source: Viruses. 2026 Apr 7;18(4):443. doi: 10.3390/v18040443 (PMC13120621; doi:10.3390/v18040443)

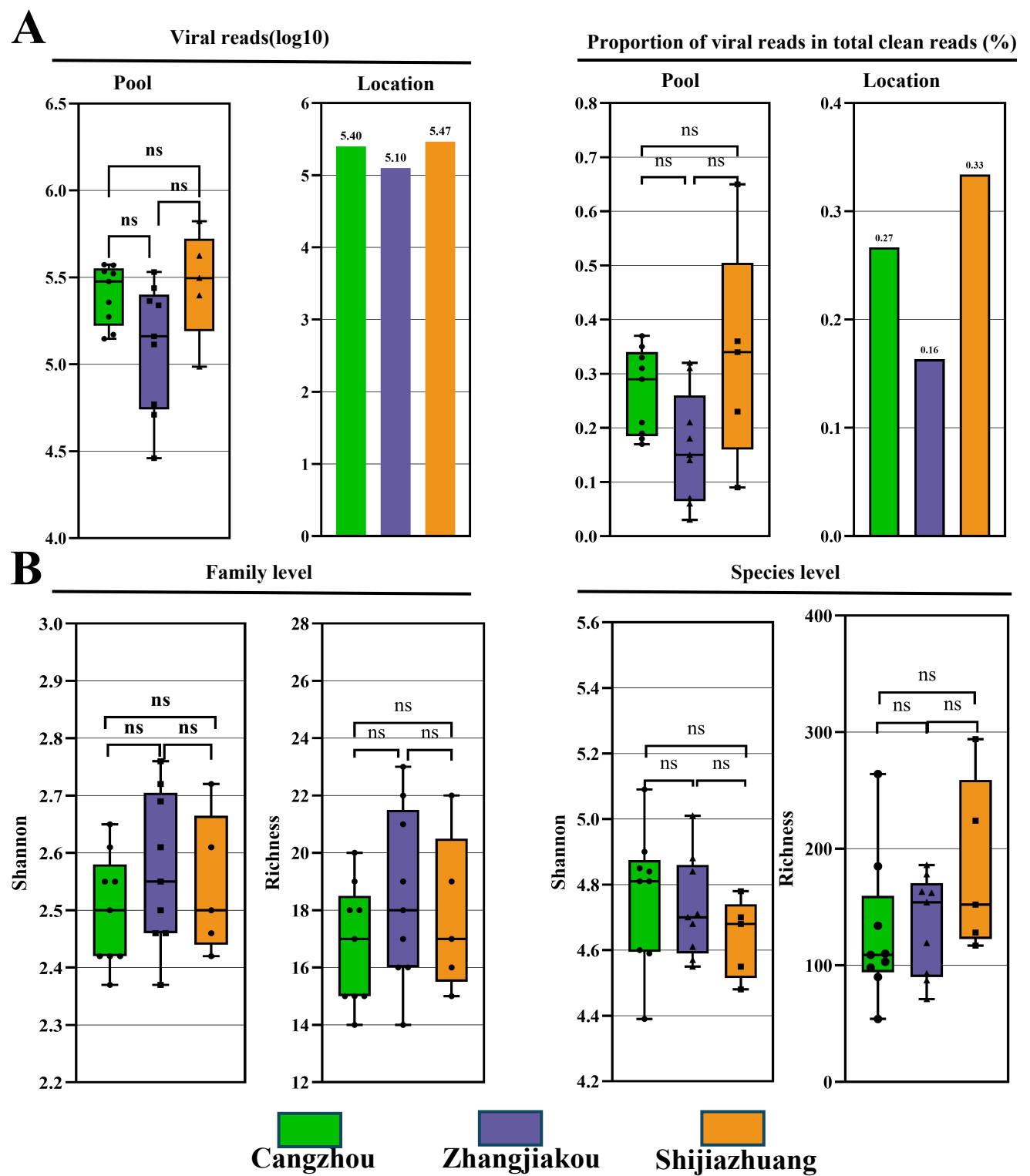

Supplement: Supplementary file 1 [file viruses-18-00443-s001.zip › Figure S1.pdf]

Bootstrap values

- >90%
- 70-90%

Tree scale 0.007

type

- B
- C
- A
- E
- F
- D

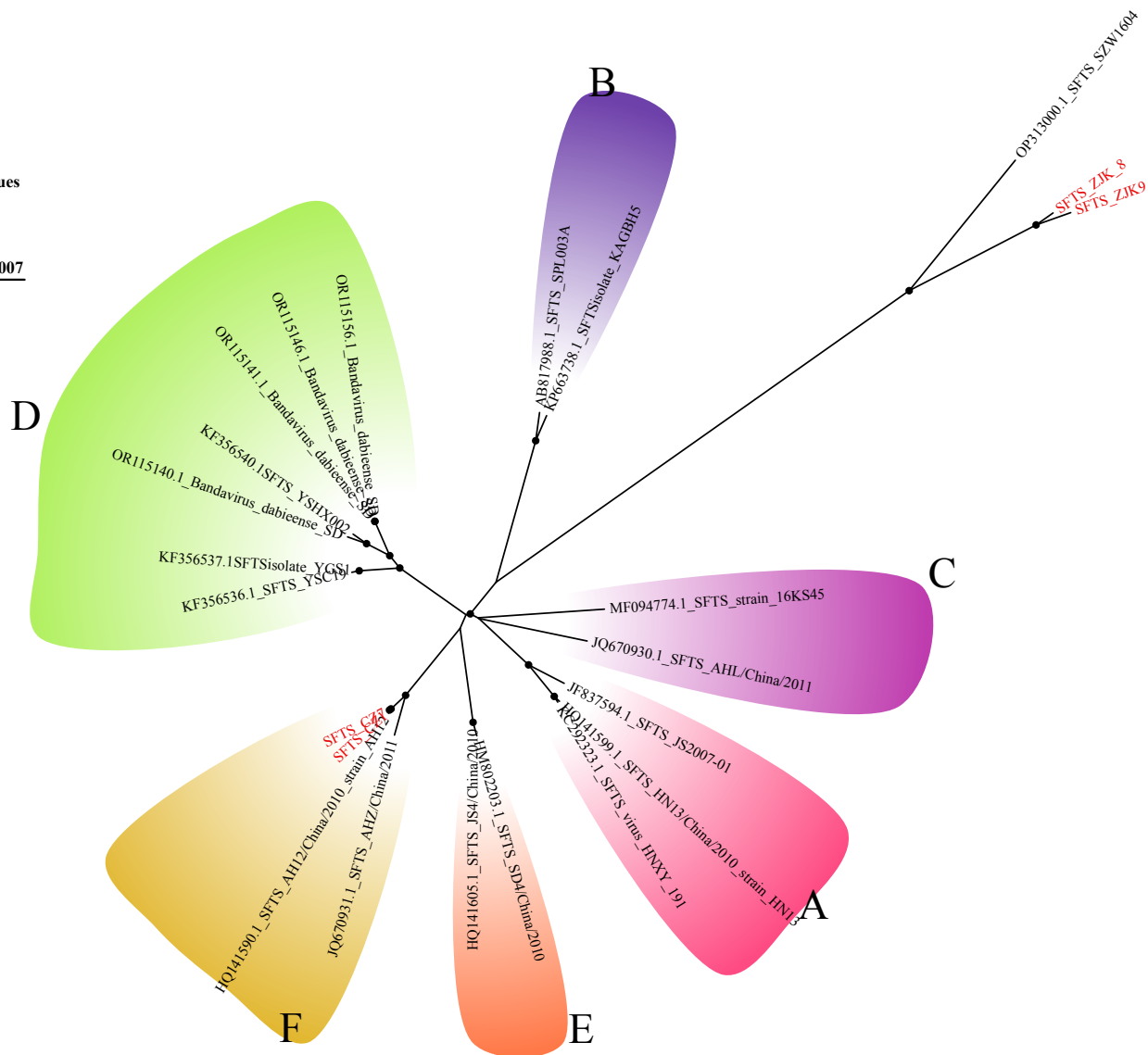

Supplement: Supplementary file 1 [file viruses-18-00443-s001.zip › Figure S2.pdf]

Bootstrap values  
● >90%  
● 70-90%

Tree scale 0.004

type

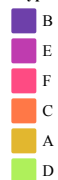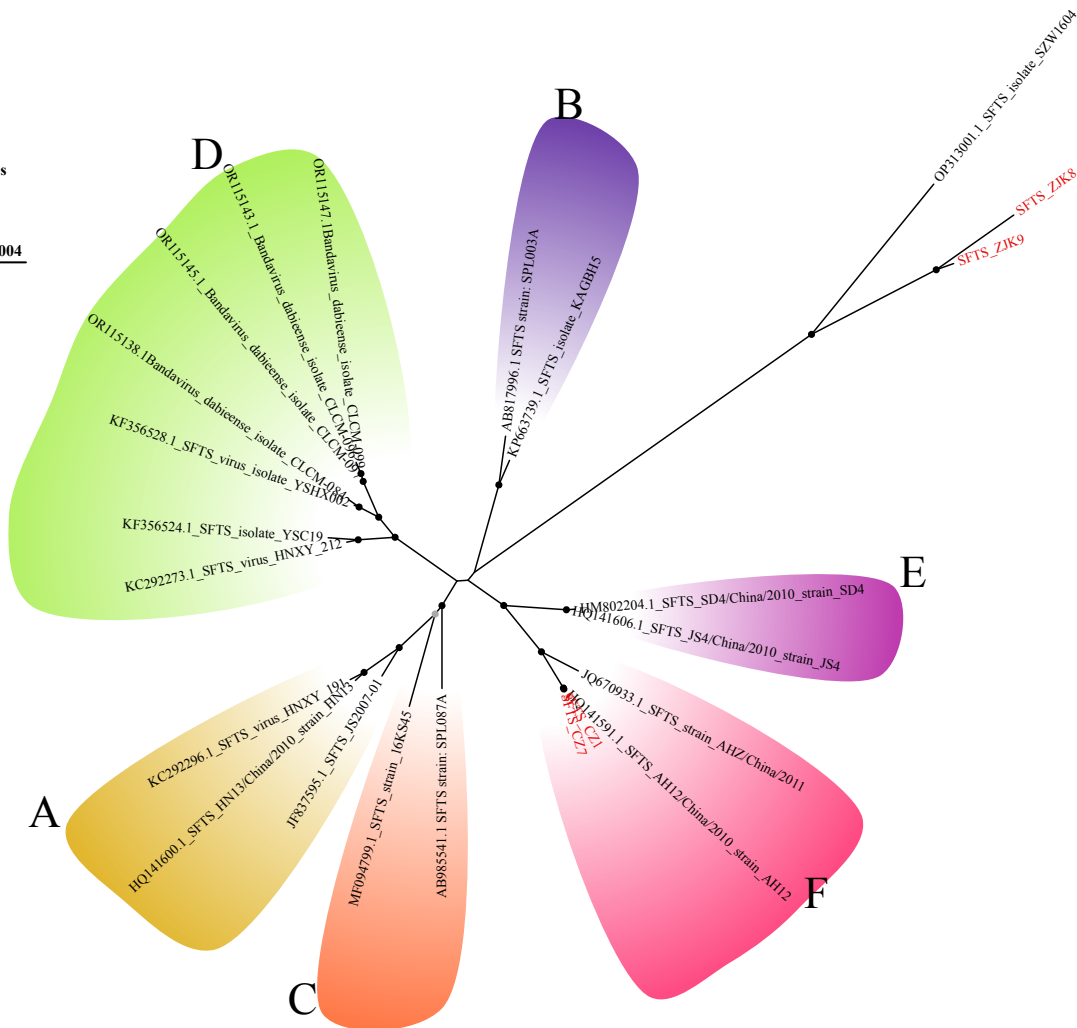

Supplement: Supplementary file 1 [file viruses-18-00443-s001.zip › Figure S3.pdf]

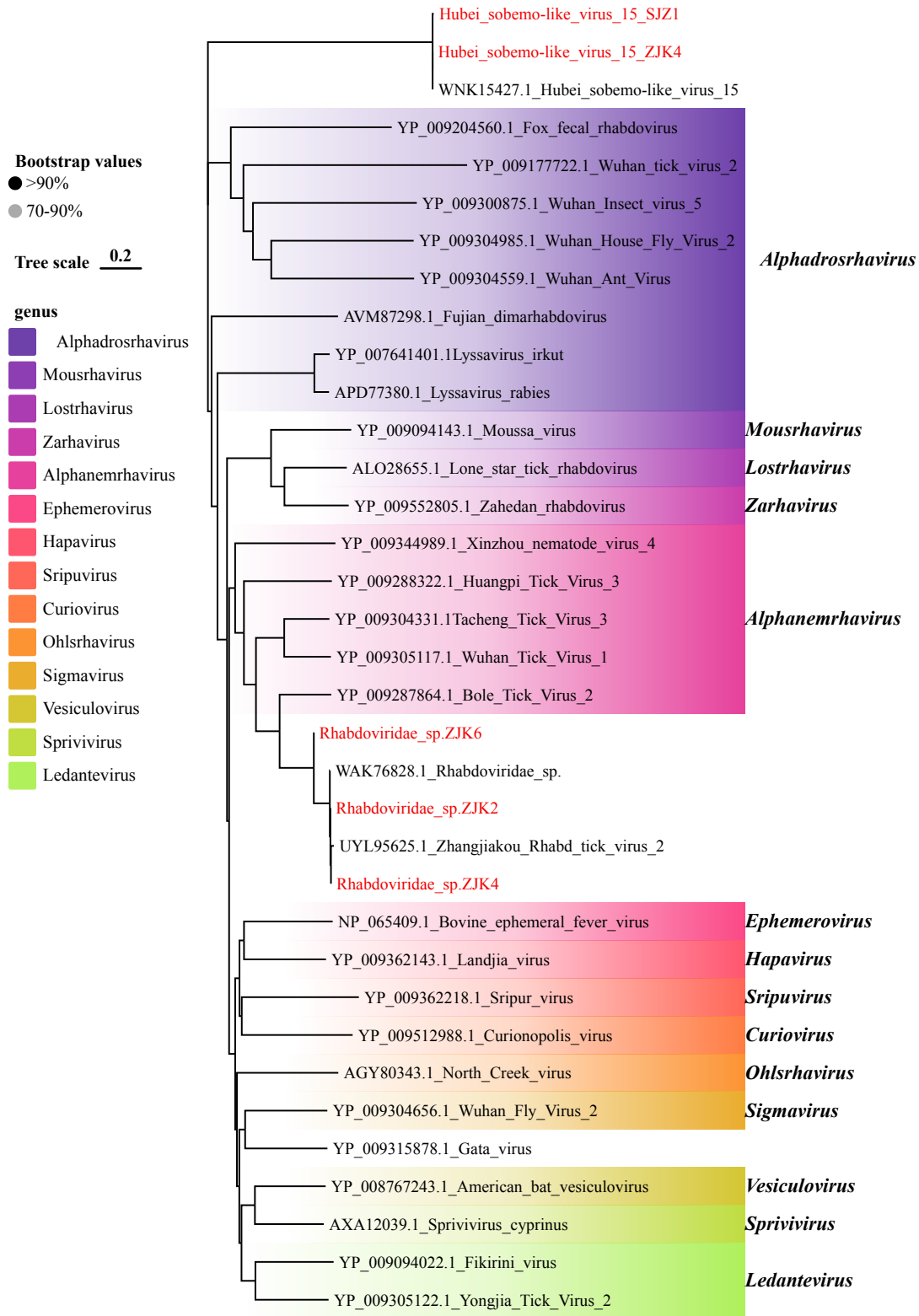

Supplement: Supplementary file 1 [file viruses-18-00443-s001.zip › Figure S4.pdf]

**Bootstrap values**

- >90%
- 70-90%

**Tree scale** 0.08

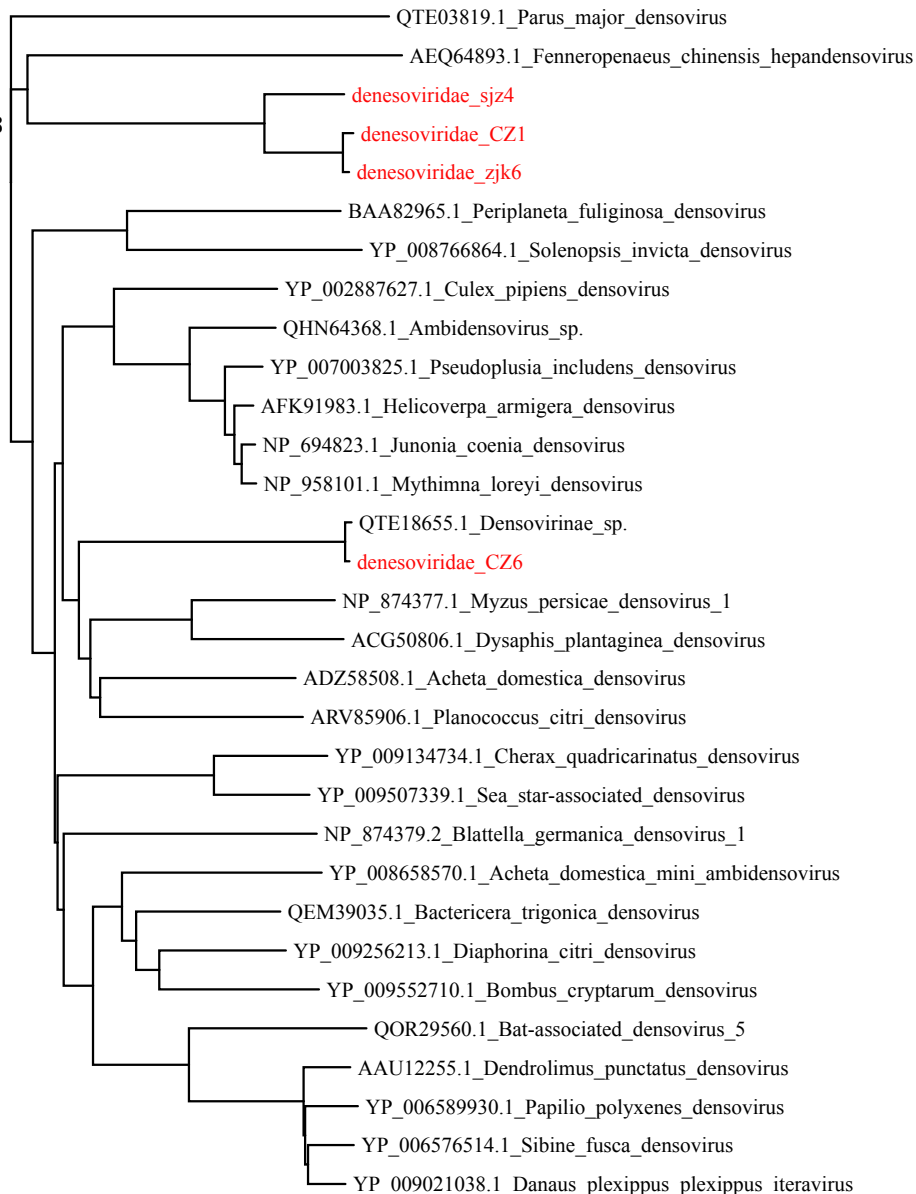

Supplement: Supplementary file 1 [file viruses-18-00443-s001.zip › Figure S6.pdf]

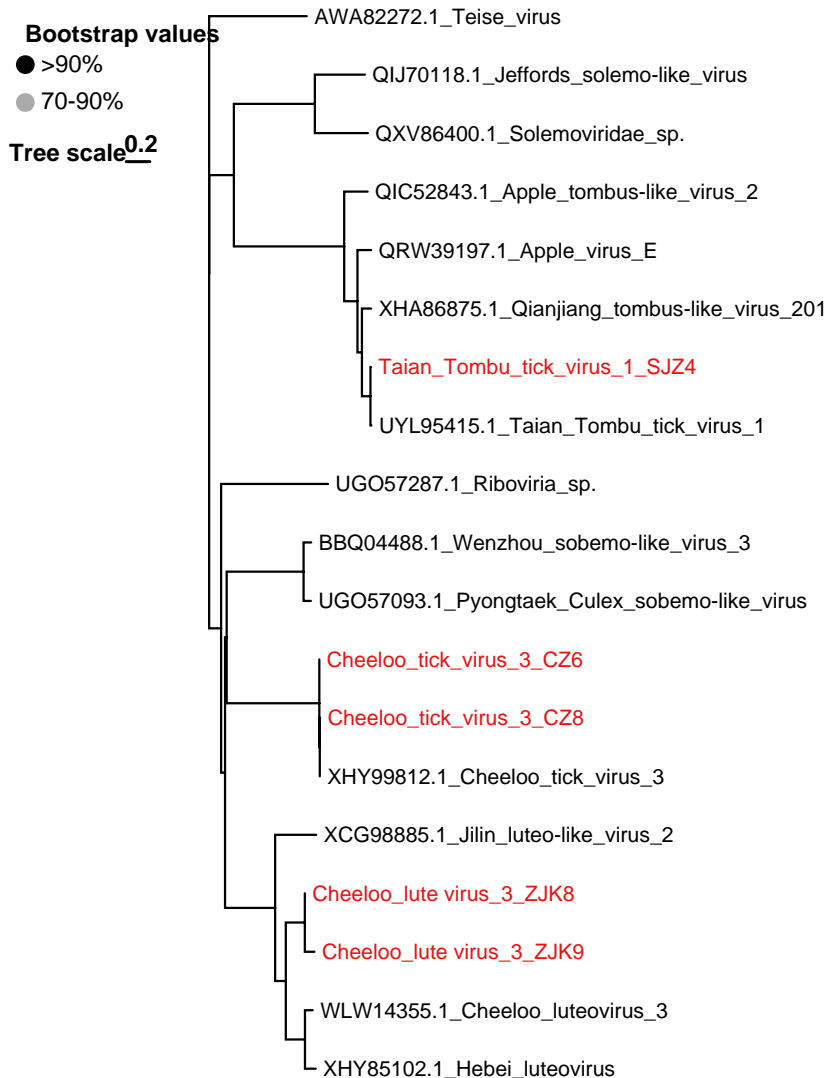

Supplement: Supplementary file 1 [file viruses-18-00443-s001.zip › Figure S7.pdf]

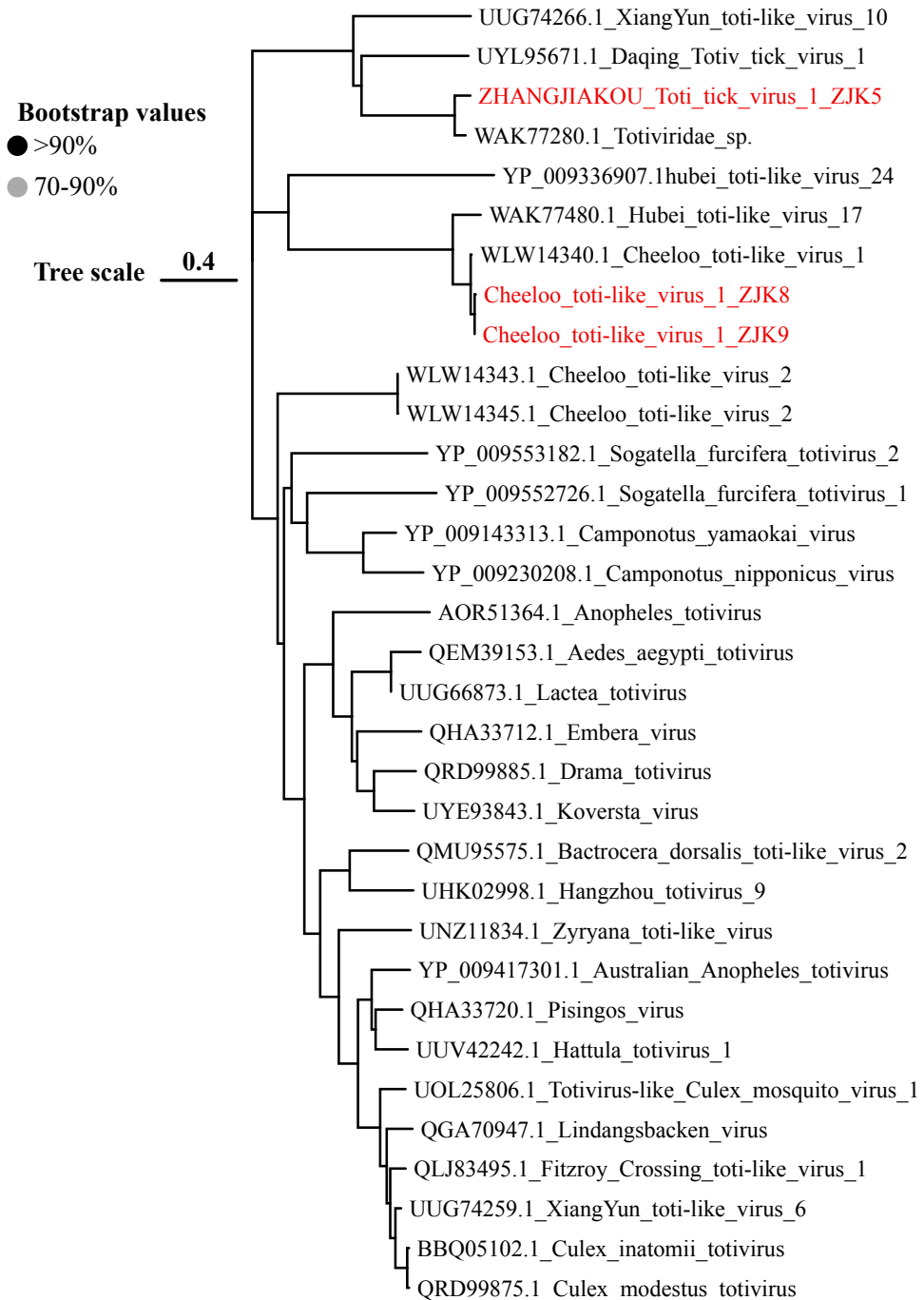

Supplement: Supplementary file 1 [file viruses-18-00443-s001.zip › Figure S8.pdf]

**Bootstrap values**

● >90%

● 70-90%

**Tree scale** 0.08

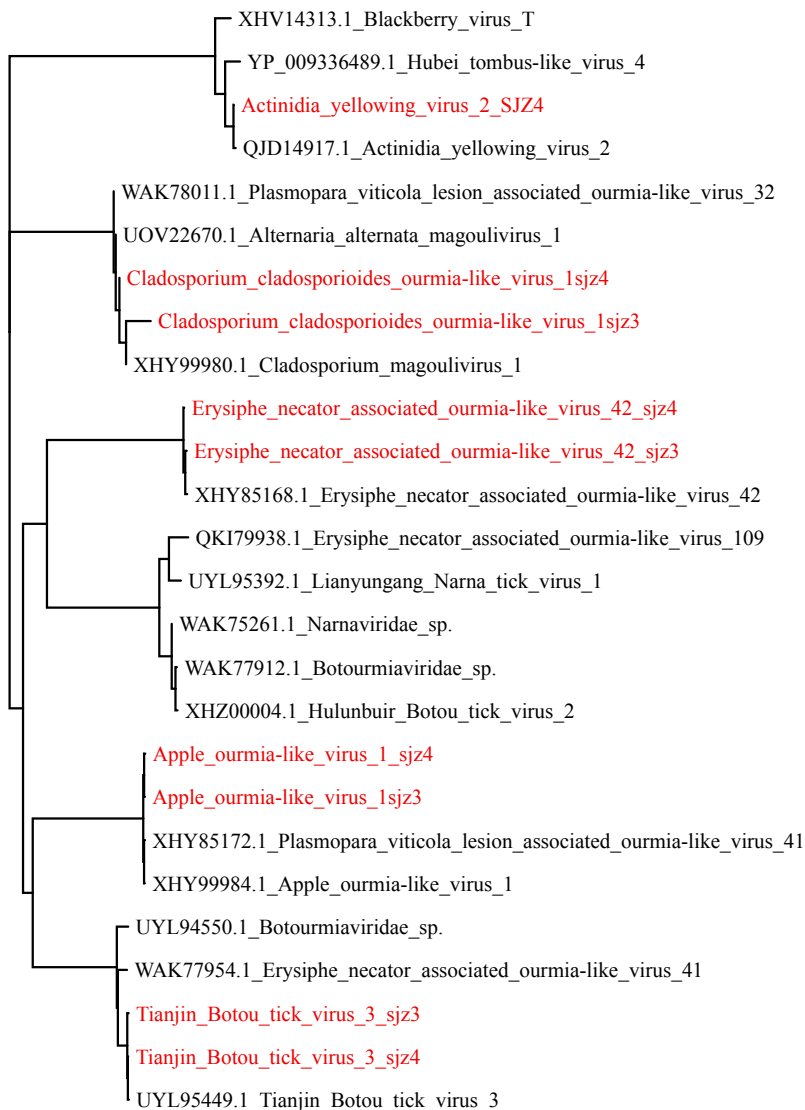

Supplement: Supplementary file 1 [file viruses-18-00443-s001.zip › Figure S9.pdf]
